# Integration of metabolomics and transcriptomics reveals the regulation mechanism of the phenylpropanoid biosynthesis pathway in insect resistance traits in *Solanum habrochaites*

**DOI:** 10.1093/hr/uhad277

**Published:** 2024-01-09

**Authors:** Meiliang Wang, Yudan Wang, Xinzhi Li, Yao Zhang, Xiuling Chen, Jiayin Liu, Youwen Qiua, Aoxue Wang

**Affiliations:** College of Life Sciences, Northeast Agricultural University, Harbin 150030, China; College of Life Sciences, Northeast Agricultural University, Harbin 150030, China; College of Life Sciences, Northeast Agricultural University, Harbin 150030, China; College of Life Sciences, Northeast Agricultural University, Harbin 150030, China; College of Horticulture and Landscape Architecture, Northeast Agricultural University, Harbin 150030, China; College of Horticulture and Landscape Architecture, Northeast Agricultural University, Harbin 150030, China; College of Life Sciences, Northeast Agricultural University, Harbin 150030, China; College of Life Sciences, Northeast Agricultural University, Harbin 150030, China; College of Horticulture and Landscape Architecture, Northeast Agricultural University, Harbin 150030, China

## Abstract

*Solanum* *habrochaites* (SH), a wild species closely related to ‘Ailsa Craig’ (AC), is an important germplasm resource for modern tomato breeding. Trichomes, developed from epidermal cells, have a role in defense against insect attack, and their secretions are of non-negligible value. Here, we found that the glandular heads of type VI trichomes were clearly distinguishable between AC and SH under cryo-scanning electron microscopy, the difference indicating that SH could secrete more anti-insect metabolites than AC. Pest preference experiments showed that aphids and mites preferred to feed near AC compared with SH. Integration analysis of transcriptomics and metabolomics data revealed that the phenylpropanoid biosynthesis pathway was an important secondary metabolic pathway in plants, and SH secreted larger amounts of phenylpropanoids and flavonoids than AC by upregulating the expression of relevant genes in this pathway, and this may contribute to the greater resistance of SH to phytophagous insects. Notably, virus-induced silencing of *Sl4CLL6* not only decreased the expression of genes downstream of the phenylpropanoid biosynthesis pathway (*SlHCT*, *SlCAD*, and *SlCHI*), but also reduced resistance to mites in tomato. These findings provided new genetic resources for the synthesis of phenylpropanoid compounds and anti-insect breeding in *S. habrochaites* and a new theoretical basis for the improvement of important traits in cultivated tomato.

## Introduction

Tomato (*Solanum lycopersicum*), as a commercially important crop, is widely planted across the world and is a popular vegetable among consumers because of the attractive color of its fruits and its distinctive sweet–sour taste, and also because it contains abundant nutrients, such as phenylpropanoids, lycopenes, and vitamins [1, 2]. However, pest occurrence in tomato cultivation significantly reduces yield and quality [3]. Tomatoes are damaged by spider mites (*Tetranychus urticae*) from the seedling stage to the fruiting stage, and in severe cases the whole leaf dries up and the inflorescence buds and fruits abscise, resulting in early senescence of plants [4, 5]. Aphids are important pests on virtually all crops, mainly concentrating on the back of leaves and tender stems to suck plant sap, resulting in yellow and curly leaves; Sustained feeding by aphids often increases the reproductive rate of the aphids, induce sooty blotch, and reduce the commerciality of fruits [6]. To date, pesticides play a critical role in controlling pests and increasing crop yields worldwide. With the extensive use of pesticides, the drug resistance of pests has gradually increased, which makes the prevention and control of pests more difficult, and even leads to food safety problems in agricultural products [7, 8]. These findings indicate that the research and development of novel, safe, and highly potent pesticides and the use of tomato self-resistance are effective ways to resist phytophagous insects such as aphids and mites.

Unlike animals or humans, plants cannot avoid the impact of environmental pressure by moving when facing a complex and changing living environment. Therefore, in the long-term evolutionary process, plants have gradually evolved a sophisticated defense approach to feeding by various insects [9, 10]. Constitutive defenses are physical or chemical factors that are inherent in the plant and are used to impede pest feeding; they are closely related to the plant genotype and serve to protect the plant throughout its life history [11]. Tomato trichomes are vectors whereby physical and chemical defenses play a common role. Plant secondary metabolites (PSMs) are synthesized and released by glandular trichomes, and include acyl sugars, terpenes, phenylpropanoids, alkaloids, and flavonoids, which can be used to directly induce or regulate defense signaling pathways to safeguard plants against phytophagous insects [12–14]. Non-glandular trichomes can hinder insect movement and decrease feeding by phytophagous insects, and significantly elevating the density and changing the type of leaf trichomes can delay the development and oviposition of insects [15]. Therefore, it is important to study the influence of constitutive defense mechanisms, such as the morphological characteristics and secondary metabolic components of tomato trichomes, on phytophagous insects.

Phenylpropanoid metabolism is one of the most important secondary metabolic pathways, yielding >8000 metabolites to date [16]. Among them, lignin is not only one of the main components of the vascular plant cell wall, but is also a natural phenolic polymer. It can combine with cellulose and hemicellulose to enhance the mechanical properties of plant cells and tissues, while forming a natural barrier against pest and disease invasion and enhancing plant resilience [17, 18]. l-Phenylalanineis formed in the shikimate pathway, and transforms into ρ-coumaroyl-CoA under the action of *PAL*, *C4H*, and *4CL*, forming the general phyenypropanoid pathway. Subsequently, ρ-coumaroyl-CoA, as the initial substrate, is transformed into lignin, flavonoids, and products of other biosynthetic pathways [19, 20]. Furthermore, phenylpropanoids and flavonoids are regulated by MYB, bHLH and WD40 transcription factors [21]. Cell walls are outposts in response to microbial and insect infestation [72]. Therefore, on the one hand the plant can promote lignification, callose accumulation, and secondary cell wall thickening, which provides a physical barrier against insect invasion; and on the other hand downstream secondary metabolites, such as flavonoids, toxic phenols, and phytoalexins, can also activate the antioxidant system and inhibit insect development and oviposition to a certain extent [22, 23]. However, only a few phenylpropanoid and flavonoid components of tomato leaves have been identified to date; in particular, most insect-resistance secondary metabolisms in tomatoes are still unknown.

Compared with a single omics tool, the rapid development of multi-omics is playing a fundamental role in the deep understanding of plant coloration and quality, responses to stress, growth, and development as well as food preservation and processing from a new biological perspective [24, 25]. Previous studies of plant defense against pest infestations have shown that the expression of *AevPAL1* and *AevTDC1* together positively regulate cereal cyst nematode resistance and interact with each other; *AevPAL1* is an important regulator of salicylic acid (SA) biosynthesis and plays a key role in resistance to cereal cyst nematode [26]. In contrast, the effects of secondary metabolites such as phenylpropanoids secreted by glandular trichomes in tomato in response to phytophagous insect infestation are poorly understood. *Solanum habrochaites* (SH) is a wild relative of the cultivated tomato, and has a variety of excellent disease and stress resistance traits [27]. The characteristics of SH are commonly used for the improvement of pest and disease resistance traits and the creation of germplasm materials in tomatoes [28]. However, when SH is used as the male parent, it can only be hybridized with cultivated tomatoes and the seed-setting rate of the resulting seeds is low [29]. It is time to adopt a multi-omics analysis strategy to explore the signaling pathways and metabolic regulatory networks of SH to lay a solid foundation for the genetic improvement of the cultivated tomato. Compared with transgenic, knockout, and antisense inhibition technology, virus-induced gene silencing (VIGS) has the advantages of a short processing cycle, low cost, and high throughput, and is therefore often used as a method to study the biological function of plant genes [30, 31]. In this study, ‘Ailsa Craig’ and SH were systematically analyzed using the metabolome combined with the transcriptome to clarify the main components and contents of phenylpropanoids and flavonoids, and to determine the biological functions of the key insect resistance genes by VIGS with a view to providing some theoretical guidance for tomato pest control and plant–insect interaction research.

## Results

### Analysis of resistance of ‘Ailsa Craig’ and *Solanum habrochaites* to aphids and mites

**Figure 1:**
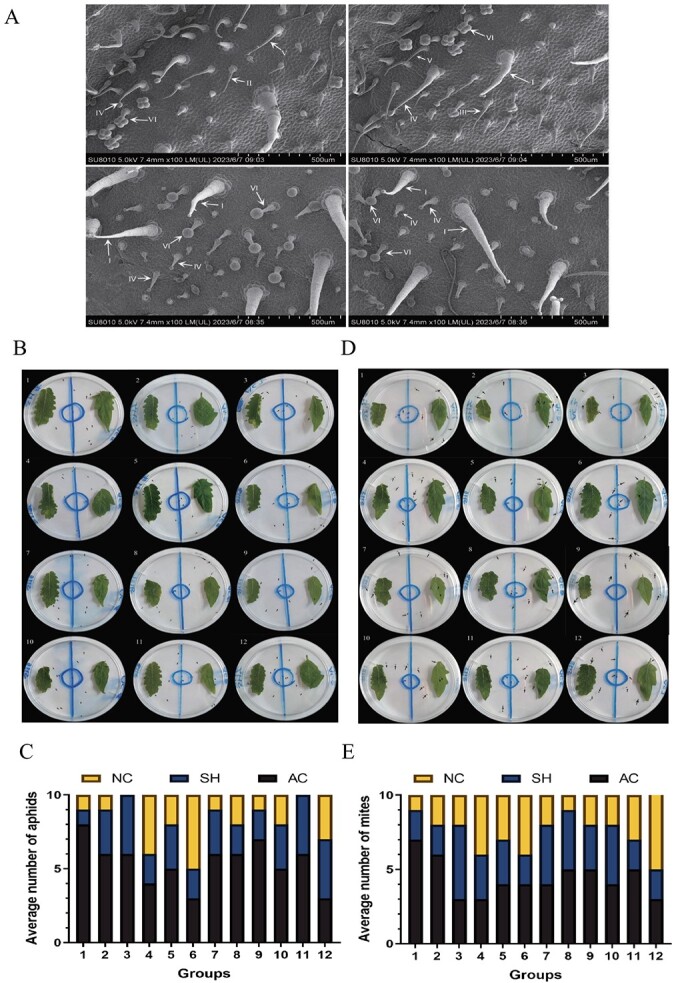
Analysis of insect resistance of tomato varieties AC and SH. **A** Cryo-SEM scans of AC and SH leaves. **B**–**E** Aphid and mite preference tests for AC and SH tomato plants. NC indicates that aphids or mites had not made a choice between AC and SH. Means and standard deviations of each group were obtained from 12 biological replicates.

Among the eight trichome types in tomato, type VI glandular trichomes are the most abundant type on the leaves and contribute significantly to insect resistance, especially in wild species [32, 33]. Cryo-scanning electron microscopy (cryo-SEM) results showed that there were abundant trichomes of types I, II, III, IV, V, and VI in AC, among which non-glandular trichomes of types II, III, and V were the most abundant. In contrast, there were types IV, V, and VI in SH. The shorter type IV and VI trichomes were the most numerous and were only found exclusively in the wild tomato, SH. Type VI trichomes in the two types of tomato had a significantly different appearance, the glandular head being smooth and round in SH and shaped like a four-leaf clover in AC; this was associated with the presence of cellular cavities in SH type VI glandular trichomes, which can accumulate large amounts of metabolites. Using fluorescence microscopy, Bergau *et al*. [32] demonstrated that type VI glandular trichomes also had four glandular heads in SH and that there was a significantly enlarged cellular cavity between the heads, which was an important storage site for metabolites secreted by glandular cells. In contrast, type VI trichomes of AC either had no cellular interstices or very small intercellular spaces that could barely store metabolites. Such a difference in type VI glandular trichomes can clearly explain why SH can accumulate more secondary metabolites than AC, thus contributing to the increased resistance of SH to phytophagous insects (Fig. 1A).

Based on the above experimental results, pest preference experiments were used to assess the relative preference of phytophagous insects for AC and SH. Ten adult aphids or mites were carefully transferred into a circle of 1 cm diameter in a Petri dish. One hour after the start of the experiment, we counted the number of aphids and mites moving to the leaves of AC or SH and those that did not make a choice (NC). The results showed that only a few pests did not make a selection, suggesting that aphids or mites had a significant preference for AC. The number of aphids or mites that preferred the leaves of SH was lower than the number preferring AC. In addition, SH had more obvious characteristics of resisting aphids than of resisting mites. Taken together, the above evidence suggested that, compared with SH leaves, with higher trichome density, phytophagous insects preferred to feed on AC leaves with lower trichome densities. However, Zhang *et al*. [65] reported that plants can secrete or synthesize volatile metabolites to attract predators or respond to infestation by phytophagous insects. We further speculated that when plants are subjected to sustained phytophagous insect feeding and damage, volatiles such as lignin, cellulose, alkali, callose, and phenolic aldehydes are released to repel phytophagous insects, attract predators, or promote communication between leaves or plants. Therefore, it is of great significance to make a profound study of the role played by trichomes in the process of insect resistance, and a novel insect resistance strategy is proposed to provide a theoretical basis for actual production practice (Fig. 1B–D).

**Figure 2:**
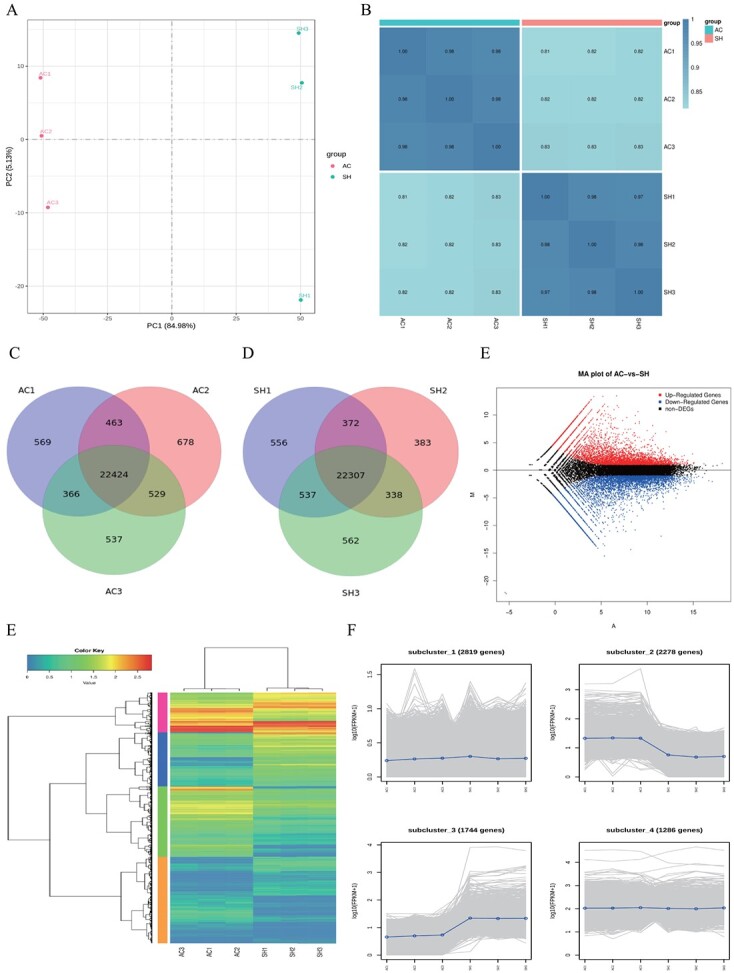
Overall transcriptome analysis and DEG analysis of both varieties. **A** PCA score plot for six samples. **B** Heat map analysis of correlation of gene expression levels among the six samples. **C**, **D** Venn diagrams of unigenes expressed in AC (**C**) and SH (**G**). **E** MA (Minus-versus-Add) plots of AC and SH. Each point represents a detected DEG. **F**, **G** Heat map analysis of DEG clustering and log_10_(FPKM+1) fold plot.

### Transcriptomic analysis in ‘Ailsa Craig’ and *Solanum habrochaites*

After removing the low-quality data, ~32 483 081 clean reads were obtained; the Q20 value was >97%, the Q30 value was >92%, and the mean GC content was 42%, signifying that the quality of the transcriptome data was relatively high and the percentage of the generated sequencing reads successfully matched to the genome was >70%.

**Figure 3:**
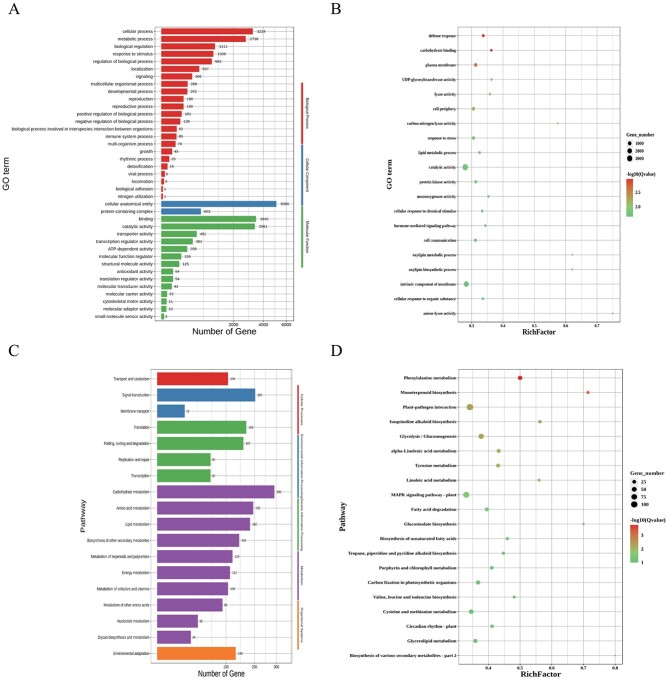
Functional annotation of DEGs in AC and SH. **A**, **B** GO classification histogram and enrichment bubble plot of DEGs in AC and SH. **C**, **D** DEGs in AC and SH were analyzed for KEGG enrichment.

Principal component analysis (PCA) was performed to investigate observed differences in two insect-resistant tomatoes, AC and SH. The PCA results showed that PC1 had an 84.98% interpretation rate, which could clearly distinguish the two tomato varieties, AC and SH (Fig. 2A). In addition, the correlation analysis showed that the correlation between the biological replicates of the samples within the group was relatively high, while there was a significant difference in the replicates between the groups of samples (Fig. 2B). In AC tomato plants, 569, 678, and 537 genes were shared between AC1 and AC2, AC2 and AC3, and AC2 and AC3, respectively. A similar situation existed in SH tomato plants, where 556, 383, and 562 genes were found in SH1 and SH2, SH2 and SH3, and SH1 and SH3, respectively (Fig. 2C and D). Using |log_2_(fold change)| > 1 and false discovery rate (FDR) < 0.05 as filtering criteria for differentially expressed genes (DEGs), a total of 9364 significant DEGs were observed in AC and SH. Among them, 4485 DEGs were visually observed to be upregulated and 4879 DEGs were downregulated (Fig. 2E). Hierarchical cluster analysis showed that there were 8127 DEGs classified into four comparison groups and each group showed similar expression trends, with a higher number of enriched clusters 1 and 2, upregulated gene expression in cluster 3, and downregulated gene expression in cluster 2 (Fig. 2F and G).

### Differentially expressed gene identification and enrichment analyses

All DEGs were annotated in Biological Process, Molecular Function, and Cellular Component. Among them, biological processes were significantly enriched in the GO classification, with cellular processes (3224) and metabolic processes (2738) dominating this category. TopGO analysis further revealed that the biological process terms ‘response defense’ (GO:0006952), ‘lipid metabolic process’ (GO:0006629), and ‘response to stress’ (GO:0006950), the molecular function term ‘kinase activity’ (GO:0016301), and the cellular component terms ‘plasma membrane’ (GO:0005886), ‘integral component of membrane’ (GO:0016021), and ‘cell periphery’ (GO:0071944) were among the most highly enriched terms (Fig. 3A and B). Based on KEGG enrichment analysis, all DEGs were annotated in Cellular Processes, Environmental Information Processing, Genetic Information Processing, Metabolism, and Organic Systems, in which most genes were concentrated in biochemical metabolic pathways. The top 20 enriched metabolic pathways showed that DEGs were enriched mainly in phenylalanine metabolism (ko00360) and monoterpenoid biosynthesis (ko00902), and plant–pathogen interaction (ko04626), isoquinoline alkaloid biosynthesis (ko00950), and glycolysis/gluconeogenesis (ko00010) (Fig. 3C and D).

**Figure 4:**
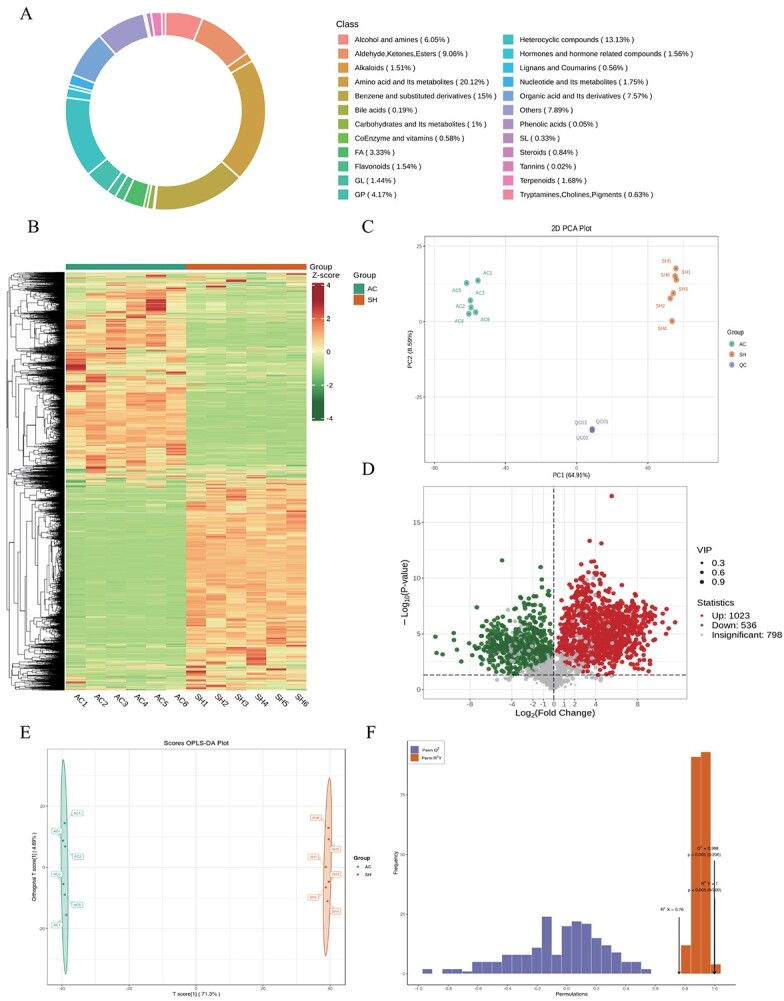
Overall metabolome analysis and DAM analysis of two tomato varieties. **A** Twelve sample metabolite categories make up the ring diagram. **B** PCA for each group of samples. **C** Overall clustering diagram of the samples. Red represents high content, green represents low content. **D** Volcano diagram of differential metabolites. Each point in the volcano diagram represents a metabolite; green points represent downregulated DAMs, red points represent upregulated DAMs, and gray points represent metabolites detected but not significantly different. **E**, **F** OPLS-DA score plot and validation plot for 12 samples.

Notably, phosphoenolpyruvate and erythritose-4-phosphate produced through the pentose phosphate pathway (*PPP*) and glycolysis pathways participate in the shikimate pathway, which in turn forms phenylalanine [35]. The common precursors of terpenoids, isopentenyl pyrophosphate (*IPP*) and dimethylallyl pyrophosphate (*DAMPP*), are derived from the MEP (Methylerythritol phosphate) pathway in plastids and the MVA (Mavalonic acid) pathway in the cytoplasm, respectively, and are converted from the intermediates of acetyl CoA or glycolysis. Therefore, the glycolysis/gluconeogenesis, phenylalanine metabolism, and monoterpenoid metabolism pathways are closely linked and are all known plant endogenous defense pathways [36, 37]. Genes in these pathways are indispensable for participation in defense and secondary metabolic processes in tomatoes.

### Classification and multivariate statistical analysis of metabolites

Non-targeted LC–MS analysis was performed on two varieties of tomato, AC and SH, and these metabolites were classified into 24 categories in positive ion mode, mainly amino acids and their metabolites (20.12%), benzene and substituted derivatives (15%), heterocyclic compounds (13.13%), and other metabolites (Fig. 4A). PC1 (64.91%) and PC2 (8.59%) cumulatively described a 79.51% level of metabolomic differences between the two tomato varieties. The above results showed that the two tomato varieties were clearly separated and the biological replicates of each variety were tightly clustered together, indicating that the experimental reproducibility was reliable, and there were significant differences between AC and SH. The hierarchical clustering results were also clearly divided into two groups, indicating that there were significant differences in metabolite content between the two varieties (Fig. 4B and C).

### Identification of differentially accumulated metabolites

A volcano diagram was constructed based on VIP > 1 and *P*-value <0.05 to identify differentially accumulated metabolites (DAMs) between AC and SH, and a total of 1102 DAMs were screened, the number of upregulated DAMs (759) being greater than the number of downregulated DAMs (343) (Fig. 4D). To further detect the reliability of the metabolomics data, a relational model discriminating the sample groups was subjected to orthogonal partial least-squares discriminant analysis (OPLS-DA). The *R*^2^X(cum) was 0.76 (+) and 0.798 (−), which indicated that the model was reliable. Subsequently, the quality of the model was examined using 200 response permutation tests, suggesting that the model was not overfitted (Fig. 4E and F).

**Figure 5:**
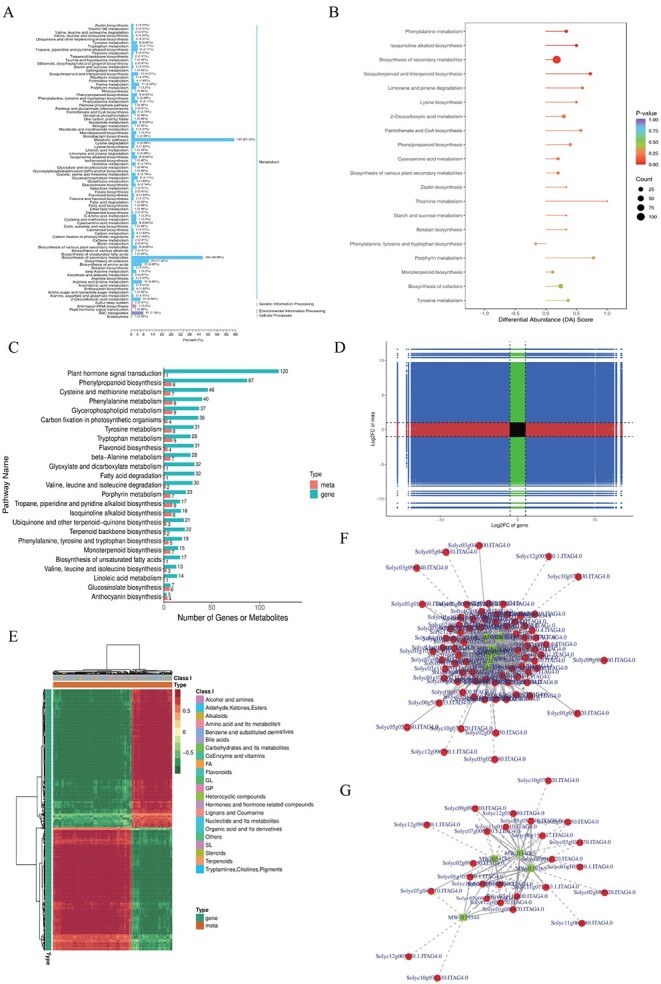
Differential metabolite KEGG enrichment analysis and bimomics integration analysis. **A** Differential metabolite KEGG classification map. **B** Differential metabolite KEGG enrichment map. **C** KEGG enrichment analysis bar graph. **D** Nine-quadrant diagram of correlation analysis. The differential multiplicities of substances with Pearson correlation coefficients >0.80 and *P*-values <0.05 in each differential subgroup were distributed into quadrants 1–9, from left to right and from top to bottom, using black dashed lines, in that order. **E** Heat map of correlation clustering of DEGs and DAMs. **F**, **G** Network diagram of the correlation between phenylpropanoid biosynthesis and flavonoid biosynthesis. Metabolites are marked with green squares and genes with red circles in the diagram. Solid lines represent positive correlations and dashed lines represent negative correlations.

Based on KEGG enrichment analysis, all DAMs were annotated in Metabolism (87.21%), Genetical Information Processing (4.11%), Environmental Information Processing (8.22%), and Cellular Processes (0.46%); among them, metabolic pathways accounted for 67.12% and secondary metabolism for 46.58% (Fig. 5A). The top 20 metabolic pathways were significantly enriched in phenylalanine metabolism (ko00360), isoquinoline alkaloid biosynthesis (ko00950), synthesis of secondary metabolites (map01110), sesquiterpenoid and triterpenoid biosynthesis (ko00909), and other metabolic pathways (Fig. 5B).

### Integrative analysis of transcriptome combined with metabolome

Correlation analysis of the transcriptome and metabolome is an experimental method to realize the full-spectrum analysis of genes and metabolites. In detail, transcriptomics is the study of gene function and gene structure at a holistic level, revealing the molecular mechanisms involved in specific biological processes and disease development, while metabolites are the end products of gene transcription–translation and are the bridge between genes and phenotypes. The combined analysis of multi-omics can preliminarily explore phytophagous insect resistance in tomatoes from two aspects: cause and effect [38, 39].

With Pearson correlation coefficient (PCC) value ≥0.8 and *P*-value ≤ 0.05 as the screening criteria, the significantly enriched metabolic pathways were identified, and these pathways mainly included the plant hormone signal transduction (ko04075), phenylpropanoid biosynthesis (ko00940), phenylalanine metabolism (ko00360), and flavone biosynthesis (ko00941) pathways. Among them, only the phenylalanine metabolism (ko00360) pathway was extremely significantly enriched (*P* < 0.01) (Fig. 5Cand E). Only the correlations detected with PCC value ≥0.8 were selected to generate nine-quadrant diagrams, and phenylpropanoid biosyntheses involving genes and metabolites with a positive correlation were located in quadrants 3 and 7 (Fig. 5D). Notably, some common DEGs and DAMs were also identified in the flavonoid biosynthesis pathway, which may be because the phenylpropanoid biosynthesis pathway was the source of precursors for flavonoid biosynthesis, and the phenylpropanoid secondary metabolism pathway played a major defense role against phytophagous insect infestation, while flavonoid biosynthesis played a secondary defense role [40]. Therefore, we speculated that the tomato response to phytophagous insect feeding stress was regulated by the co-expression of DEGs and DAMs related to phenylpropanoid and flavonoid biosynthesis. Correlation network analysis was performed to investigate the gene and metabolite regulatory mechanism of phenylpropanoid and flavonoid biosynthesis in tomatoes (Fig. 5F and G).

### Analysis of differentially expressed genes and differentially accumulated metabolites related to phenylpropanoid and flavonoid biosynthesis pathways for insect-resistance in ‘Ailsa Craig’ and *Solanum habrochaites*

**Figure 6:**
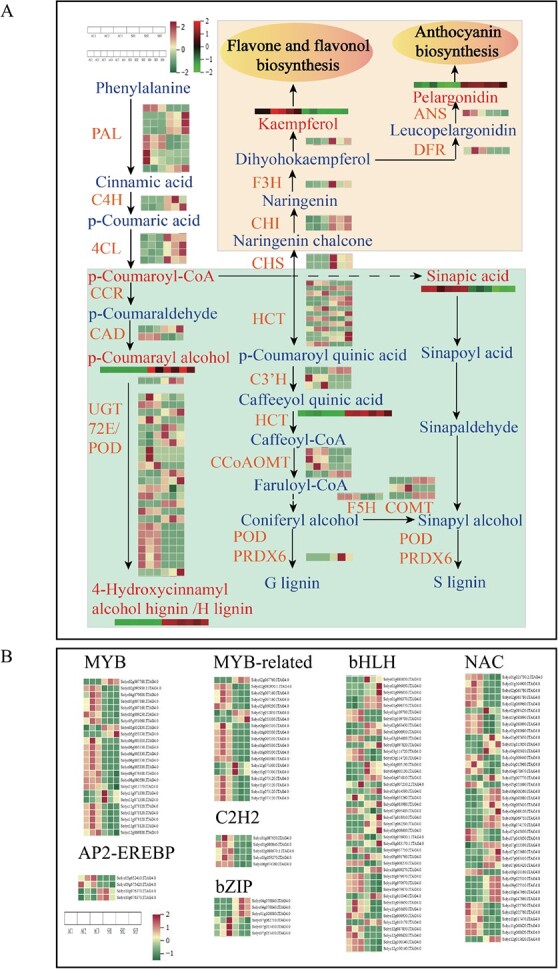
Phenylpropanoid and flavonoid biosynthesis network diagram in tomatoes of two cultivars. **A** Phenylpropanoid and flavonoid biosynthesis pathways in AC and SH. Gene expression is shown according to the mean FPKM of three biological replicates; green indicates low expression and red indicates high expression. High-abundance metabolites are indicated in dark red and low-abundance metabolites in dark green. PAL, phenylalanine ammonialyase; C4H, cinnamate 4-hydroxylase; 4CL, 4-coumaroyl CoA ligase; CCR, cinnamoyl-CoA reductase; HCT, shikimate O-hydroxycinnamoyltransferase; CAD, cinnamyl-alcohol dehydrogenase; UGT72E, coniferyl-alcohol glucosyltransferase; CCoAOMT, caffeoyl-CoA O-methyltransferase; C3H, 5-O-(4-coumaroyl)-d-quinate 3′-monooxygenase; POD, peroxidase; COMTcaffeic acid 3-*O*-methyltransferase; F5H, ferulate-5-hydroxylase; CHS, chalcone synthase; CHI, chalcone isomerase; DFR, dihydroflavonol-4-reducatse; F3H, flavanone 3 β-hydroxylase; FLS, flavonol synthase; FNS, flavone synthase; ANS, anthocyanidin synthase. **B** Expression patterns of the main transcription factors in the comparison of AC and SH.

Based on the reported biosynthesis pathways of phenylpropanoids and flavonoids in model plants, we constructed a regulatory network diagram of DEGs and DAMs in tomatoes (Fig. 6A). In total, 8 DAMs were significantly correlated with 87 DEGs in the phenylpropanoid biosynthesis pathway, and 4 DAMs were significantly correlated with 31 DEGs in the flavonoid biosynthesis pathway. Phenylalanine, as a precursor for phenylpropanoid biosynthesis, was generated by the shikimate pathway, which constituted a phenylpropane pathway generated in response to early biotic stress under regulation by key rate-limiting enzymes *PAL*, *C4H*, and *4CL*. In the early defense stage of SH, the upregulation of four *PAL*s (Solyc00g500353, Solyc03g042560, Solyc05g056170, and Solyc10g086180), two *C4H*s (Solyc05g047530 and Solyc06g150137) and four *4CL*s (Solyc02g088710, Solyc03g111170, Solyc03g117870, and Solyc08g076300) had higher expression levels but their expression was lower in AC than in SH. The above data clearly showed that, compared with AC, SH can efficiently induce and activate the plant phenylpropanoid common pathway to form a complete early defense network, thus contributing to SH resisting invasion by phytophagous insects.

Specifically, in the phenylpropanoid biosynthesis pathway, *HCT* was located upstream and downstream of *C3′H*, which was the key fulcrum for the synthesis of H-, G- and S-lignin, and determined the carbon source flow direction of lignin units [41, 42]. A total of 13 *HCT*s were identified, and 6 and 7 were highly expressed in AC and SH, respectively. *COMT* can catalyze the methylation of caffeic acid to synthesize ferulic acid, and then promotes the accumulation of sinapic acid. Two *COMT*s were upregulated and one *COMT* was decreased in SH. Three *CCoAOMT*s (Solyc02g093230, Solyc02g093250, and Solyc09g082660) and one *CCoAOMT* (Solyc10g050160) were highly expressed in AC and SH tomatoes, respectively. Reducing the expression level of *CCoAOMT* will reduce the accumulation of lignin and have a negative influence on plant growth [43]. *CAD*, *UGT72E*, and *E1.11.1.7* were the key enzyme systems involved in the final step of the synthesis pathways of the three lignin monomers [44]. *CAD* catalyzed the generation of ρ-coumaryl alcohol, and then promoted the accumulation of 4-hydroxycinnamyl alcohol 4-d-glucoside through *UGT72E* cascade catalysis [45]. In both AC and SH, there was high expression of genes that can catalyze *CAD* enzymes, solyc02g069250 and solyc02g030480, respectively; however, only one gene (solyc02g063000) in SH was identified to catalyze *UGT72E*. Thus, lignin monomers synthesized in the cytoplasm and passing through the cell membrane polymerized in the cell wall, catalyzed by *E1.11.1.7*, and determined the structure of lignin-based polymers to a certain extent. Compared with AC, nine *E1.11.1.7* was expressed at a higher level in SH leaves, and only the expression of *E1.11.1.7* (solyc02g092580) was significantly upregulated, which suggested that *POD* can effectively remove the OH^−^ and H_2_O_2_, which are harmful to cells in plants, promoting the lignification of wounds after phytophagous insect feeding, and increasing the thickness of cell layer.

In the flavonoid biosynthetic pathway, *CHS* was considered to be the key enzyme controlling the metabolic direction of flavonoids [109]. One *F3H* (solyc02g083860), two *CHS*s (solyc09g091510 and solyc12g0980990.1), two *CHI*s (solyc05g010320 and solyc05g052240), and one *FLS* (solyc11g013110) were only expressed at a higher level in SH, indicating that SH had a strong secondary metabolism of flavonoids. Plant color affected insect pollination and the ornamental value of plants [46]. In the anthocyanin biosynthesis pathway, the accumulation of pelargonidin also enhanced the insect resistance of SH to some extent.

Eight hundred and eighty-three transcription factors (TFs) were identified and classified into 37 families. The MBW complex, containing *MYB*, *bHLH*, and *WD40*, was a key regulation factor widely involved in plant defense responses and can directly regulate downstream structural genes of phenylpropanoid biosynthesis [47]. A total of 24 *MYB*s, 19 *MYB-related*s, and 42 *bHLH*s were identified to play an important role in metabolic pathways, and, compared with AC, several TFs were very significantly upregulated in SH (*P* < 0.01), such as one *MYB* (solyc05g053330) and three *bHLH*s (solyc10g009270, solyc07g018010, and solyc03g097820), which may regulate structural genes involved in plant defense responses to phytophagous insects. Additionally, previous studies illustrated that other TFs were identified in AC and SH that can be involved in the regulation of defense responses against phytophagous insects, including 41 *NAC*s, 6 *bZIP*s, 5 *C_2_H_2_*s, and 4 *AP2-EREBP*s [48, 49] (Fig. 6B).

Peng *et al*. [50] demonstrated that the SA signaling pathway played an important role in plant defense responses to phytophagous insects, as evidenced by a significant increase in the accumulation of H_2_O_2_, the content of endogenous SA, and the expression of key genes involved in SA biosynthesis in tomato seedlings. SA was synthesized in plants by two pathways, the phenylalanine (*PAL*) and the isochorismate (*ICS*) pathways, both of which require shikimate pathway-derived chorismate as precursors. *NONEXPRESSOR OF PATHOGENESIS-RELATED GENES* 1 (*NPR*1) was a key regulator in the plant systemic acquired resistance (SAR) signaling process, located downstream of the SA synthesis pathway, and interacted with transcription factors, such as *TGA*, to activate the production of many resistance-associated proteins, including pathogenesis-related (*PR*) protein [51, 52]. Understanding the changes in the expression of these SA pathway-related genes may provide effective measures for exploring a wide range of insect pests and adversity stresses experienced during tomato production. Thus, spraying tomato plants with solutions containing either SA or methyl salicylic acid (Me-SA) or regulating genes related to the SA synthesis pathway can significantly increase H_2_O_2_ levels and improve the defense response of tomato plants against phytophagous insects [53].

### Phenylpropanoid biosynthesis was induced by mites

**Figure 7:**
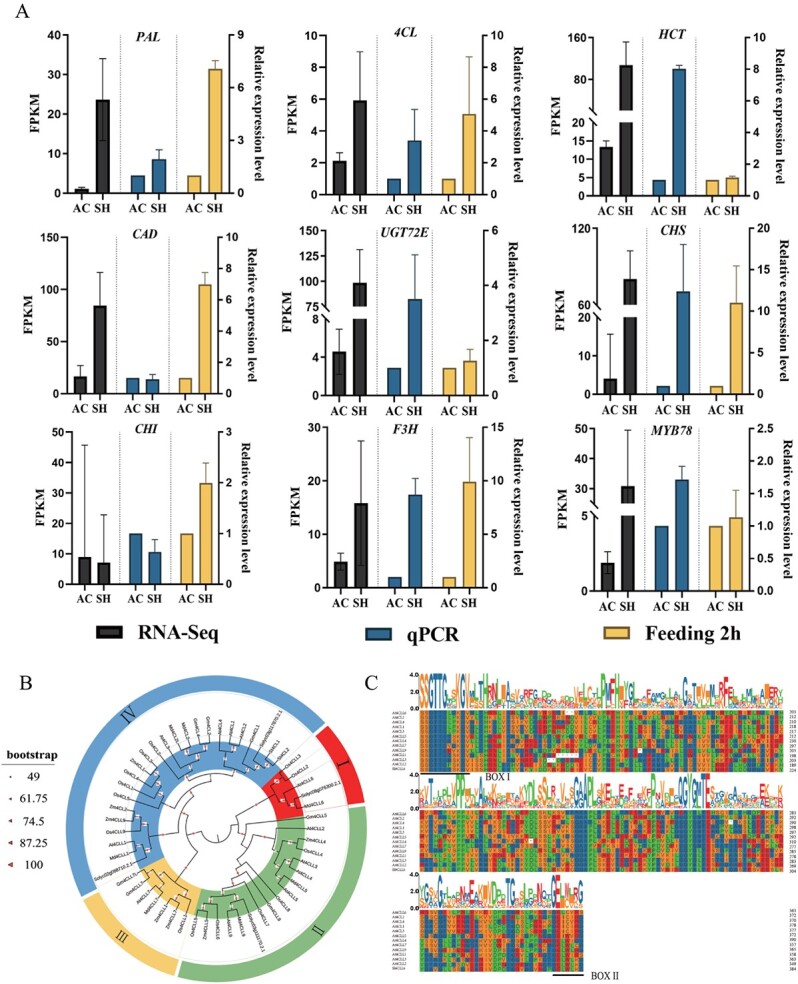
qRT–PCR validation of expression patterns of candidate genes and phylogenetic analysis and amino acid sequence alignment analysis of *Sl4CLL6*. **A** Comparative expression patterns of the nine genes. Panels on the left indicate transcriptome results and the middle panels indicate the qRT–PCR results. Panels on the right indicate results after feeding the mites for 2 h. The scale on the left axis indicates the FPKM value and the scale on the right axis indicates the relative expression level. Data are mean ± standard deviation of three biological replicates. **B** Phylogenetic analyses of Solyc02g088710, Solyc03g117870, Solyc03g111170, and Solyc08g076300 with proteins of *4CL*s or *4CLL*s from other species. Phylogenetic analyses were performed using MEGA 6.0 software and the proximity method. **C** Multiple sequence alignments of amino acid sequences of Solyc08g076300 with *At4CL* or *At4CLL*s, including *At4CLL6* (Q84P24.2), *At4CL2* (Q9S725.2), *At4CL4* (Q9LU36.1), *At4CL1* (Q42524.1), *At4CL3* (Q9S777.1), *At4CLL5* (Q84P21.2), *At4CLL4* (P0C5B6.1), *At4CLL7* (Q9M0X9.1), *At4CLL9* (Q84P23.2), *At4CLL1* (Q9LQ12.1), *At4CLL3* (Q3E6Y4.2), and *At4CLL2* (Q84P25.2).

We validated the reliability of the RNA-seq results, using RT–qPCR to examine the expression levels of nine candidate genes (*SlPAL*, *Sl4CL*, *SlHCT*, *SlCAD*, *SlUGT72E*, *SlCHS*, *SlCHI*, *SlF3H*, and *SlMYB78*) involved in the phenylpropanoid biosynthesis pathway. The expression patterns in AC and SH obtained by RT-qPCR were similar to those obtained by RNAseq, which suggested that transcriptome data were reliable and valid (Fig. 7A). Moreover, expression of genes involved in the phenylpropanoid biosynthesis pathway was increased after the mites fed on AC and SH leaves for 2 h, and the degrees of upregulation of *PAL*, *4CL*, *CAD*, and *CHI* were more significant in SH, suggesting that key genes in the phenylpropanoid and flavonoid biosynthesis pathways might play a regulatory role in tomato defense against aphid and mite infestation.

### Identification and sequence analysis of *Sl4CLL6* (Solyc08g076300)

 Previous studies had shown that the phyenypropanoid biosynthesis pathway in response to phytophagous insect stress was focused on the regulation of PAL, HCT and F3H, which may be due to the fact that the amount of *PAL* accumulation was significantly correlated with defense against pathogens and regulation of pest behavior [54, 55]; *HCT* was responsible for flux partitioning between C-, G-, 5H, and S-lignin [56]. In wild-type rice *ZH11* plants, *OsmiR396* inhibited the expression of the growth-regulating factor *OsGRF8*, which in turn downregulated the expression of *OsF3H* in the flavonoid biosynthesis pathway, and the flavonoid content was simultaneously decreased, whereas in plants in which *OsmiR396* was silenced the flavonoid content was significantly increased and they were more resistant to brown plant-hopper than the wild type [57]. However, it is worth noting that *4CL* was also one of the key enzymes in the phenylpropanoid metabolic pathway, which played a pivotal role in linking lignin precursors to the branching pathways and helped to direct carbon fluxes towards phenylpropanoid biosynthesis or flavonoid biosynthesis [58]. Therefore, it was of great importance to explore the function, characterization, and role of the *4CL* gene family in metabolite biosynthesis.

A phylogenetic tree was constructed to analyze the phylogenetic relationships between *4CL*s differentially expressed in AC and SH and 4CL proteins in *Arabidopsis thaliana L.*, *Solanum tuberosum* L., *Oryza sativa* L., *Malus pumila* Mill., *Glycine max* L., and *Zea mays* L. As shown in Fig. 7B, the *4CL* family was divided into four subfamilies, among which, Solyc02g088710, Solyc03g117870, and Solyc03g111170 were closely related to *At4CLL1* and *Md4CLL1*, *St4CL1* and *St4CL2*, and *At4CLL9* and *Md4CLL9*, respectively, which suggested that there may be similarity in gene function among them. Specifically, Solyc02g088710 was involved in sporopollenin biosynthesis and pollen exine formation during the period of tomato microgametogenesis [59]. Solyc03g117870 was identified as a candidate gene involved in SA signaling regulation during infestation of *Solanum lycopersicum* by the Gram-positive bacterium *Clavibacter michiganensis* subsp*. michiganensis* (CMM) [60]. Solyc03g111170 was more homologous to *At4CLL9*, and Zhao *et al*. [61] reported that in a *pagERF81*-overexpressing strain in poplar 84 K (*Populus alba* × *P. glandulosa*), *Pag4CLL9*, *PagCAD6*, and *PagCCR1* were downregulated in lignin biosynthesis pathways, which resulted in a decreased lignin content of the cell wall. While Solyc08g076300 was more homologous to *Md4CLL6*, there were fewer reports on its biological function, and only in one study [62] was it found that, compared with the germination stage, the *Sl4CLL6* gene was directly involved in the accumulation of flavonoid biosynthesis in stems, and its expression was increased 8.81-fold in the vegetative growth stage and 13.35-fold in the flowering stage, thus improving the antioxidant capacity of *Cynomorium songaricum*. In addition, based on the changes in the expression of each key gene after 2 h of feeding by mites, it was shown that the expression of *Sl4CLL6* was significantly increased 4.3-fold in SH compared with AC, which suggested that *Sl4CLL6* may play a key role in the tomato’s defense against insect pest stress.

As reported previously [39, 63, 64], *4CL* belongs to the adenylate-forming enzyme family, with the presence of a highly conserved Box I (SSGTTGLPKGV) for the AMP-binding domain and a Box II (GEICIRG) at the N-terminus. Amino acid sequence alignment of *Sl4CLL6* (Solyc08g076300) with *At4CLs* revealed that typical Box I domains (SSGTTGVGKGV) and Box II domains (GELWLCG) were also present at the N-terminus of *Sl4CLL6* (Fig. 7C). Box I was highly conserved among all adenylate-forming enzyme protein families and was responsible for catalyzing substrate recognition and binding, whereas Zhang *et al*. [65] and Yuan *et al*. [66] found that Box II was likewise highly conserved in almost all *4CL*s, with a central cysteine residue thought to be directly involved in substrate catalysis, and its protein activity can be effectively inhibited by sulfhydryl chemical inhibitors. In summary, resolving the regulatory mechanism of *Sl4CLL6* in the formation of insect resistance traits in tomato may provide a reference value for the involvement of *4CL* in metabolic and signaling response mechanisms.

### Effects of silencing *Sl4CLL6* on the phenylpropanoid biosynthesis pathway and resistance in response to *Tetranychus urticae*

**Figure 8:**
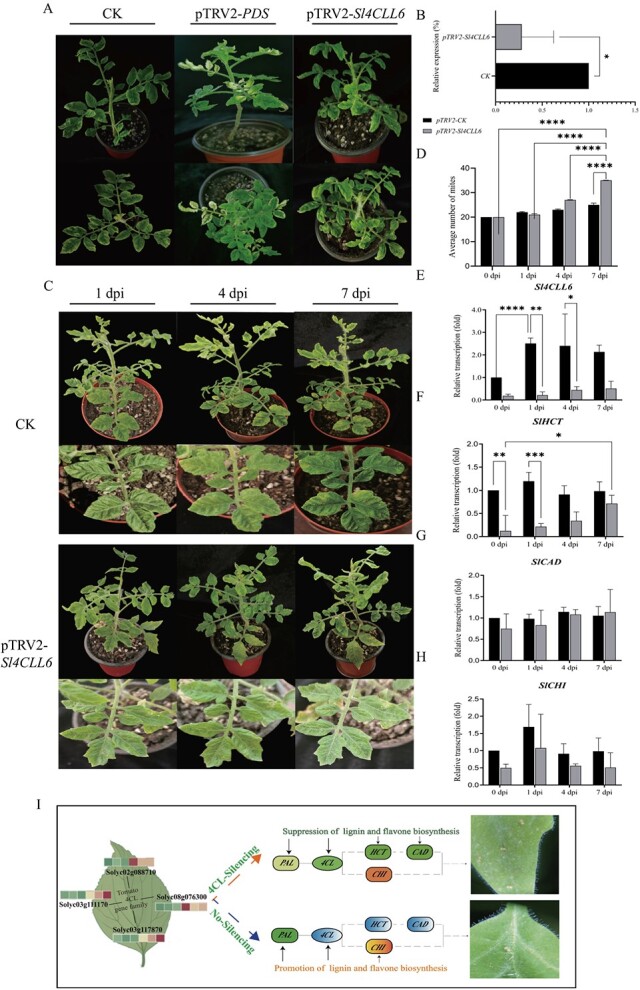
Downregulation of *Sl4CLL6* gene expression reduced resistance to mites in tomato plants. Silencing of *Sl4CLL6* by TRV-VIGS. **A** Negative (CK), *TRV2-PDS*, and *TRV2-Sl4CLL6* plants. **B** Expression of *Sl4CLL6* gene in CK and *TRV2-4CLL6* plants. **C** Changes in phenotypes of CK and silenced plants after inoculation with mites for 1, 4, and 7 days. **D** Average number of mites in CK and silenced plants after inoculation with mites for 0, 1, 4, and 7 days. **E**–**H** Changes in the expression of *Sl4CLL6* and downstream genes of the phenylpropanoid biosynthesis pathway, *SlHCT*, *SlCAD*, and *SlCHI*, in CK and silenced plants 0, 1, 4, and 7 days after inoculation with mites. **I** Potential mechanisms by which *Sl4CLL6* regulates lignin accumulation and shapes resistance to mites in tomato. Each value indicates the mean ± standard deviation of three biological replicates. ^*^, ^**^, ^***^ and ^****^ indicate significant differences between CK and silenced plants with *P* < 0.01,* P *< 0.001, *P *< 0001, *P* < 00001, respectively, as determined by *t*-test.

*Sl4CLL6* was temporarily silenced by VIGS, and *pTRV2-Sl4CLL6*-silenced plants were constructed to investigate the role of *Sl4CLL6* in phenylpropanoid biosynthesis and resistance to mite feeding. Twenty days after vacuum infestation with *pTRV2-PDS*, the plants presented obvious chlorosis and whitening symptoms, demonstrating the effectiveness of virus inoculation in silencing the plants (Fig. 8A). In addition, the transcription of *Sl4CLL6* was significantly decreased in *pTRV2-Sl4CLL6* compared with control (CK, negative plant, *TRV2*-*TRV1*) (silencing efficiency was ~71.83%), indicating that the *pTRV-VIGS* system was successfully established in AC (Fig. 8B).

When *TRV2-SlPDS* plants showed clear robust signs of photo-bleaching, leaves of *TRV2-Sl4CL* and CK plants were inoculated with mites; the infection phenotypes of tomato leaves were subsequently observed and the numbers of mites were recorded at 0, 1, 4, and 7 days post-inoculation (dpi, day post inoculation). Significant infection symptoms were also observed between the *Sl4CLL6*-silenced strain and CK during short-term exposure to *T. urticae*. Specifically, at 0, 1, and 4 dpi, there was no difference in symptoms between the silenced and CK plants, and the mites were mostly concentrated near the veins of the leaves and then extended outward to the stems. The expression levels of *Sl4CLL6* at 0, 1 and 4 dpi were 0.185, 0.215 and 0.446 in silenced plant, respectively, while they were 1, 2.506 and 2.402 in CK plant, respectively. The average number of mites was 20,22 and 23 in silenced plant, respectively, while it was 20, 21 and 27 in CK plant. At 7 dpi, the *Sl4CLL6*-silened strain showed severe symptoms of *T. urticae* infestation, with dense spots of *T. urticae* damage covering the leaves in multiple places and constituting a thin wire mesh on neighboring stems and leaves, causing large yellow spots on the leaf blade and localized visible collapse and water loss. Compared with the silenced plants, the CK plants were in better condition, and the location of the mite-infested leaves was confined to a smaller area (Fig. 8C). At this time, the expression of *Sl4CL* in CK and silenced plants was 2.135 and 0.511, respectively, and the average mite numbers were 25 and 35. The above results suggested that, compared with CK plants, the silenced plants had mites with improved adaptation and faster reproduction, as significantly demonstrated by a total number of 35 live mites on the silenced plants after 7 days of feeding. Thus, both the altered plant phenotype and mite behavior after silencing of *Sl4CLL6* suggested that *SL4CLL6* was a key insect resistance gene in the phenylpropanoid biosynthesis pathway (Fig. 8D and E).

In addition, we also examined the effect of *Sl4CLL6* gene silencing on the expression of genes downstream of the phenylpropanoid biosynthesis pathway. The expression of *SlHCT* and *SlCHI* was also significantly decreased in *pTRV2-Sl4CL*, but was still significantly lower than in CK (Fig. 8F–H). Surprisingly, the expression of *SlHCT*, *SlCAD*, and *SlCHI* also changed after feeding by mites, but their expressions were similarly significantly lower than those in CK. Specifically, the transcription of *SlHCT* in silenced plants gradually increased with the intensification of pest stress; the expression of *SlCHI* reached a maximum of 1.076 at 1 dpi and then slowly decreased, whereas the expression of *SlCAD* did not change significantly in CK and silenced plants. The above results suggested that *Sl4CLL6* not only regulated phenylpropanoid biosynthesis in response to feeding by mites, but was also regulated by a cascade of multiple genes in the downstream part of the phenylpropanoid biosynthesis pathway (Fig. 8I).

## Discussion

Tomato, as a popular economic crop with high nutritional value, is the most widely studied dicotyledonous model plant besides *A. thaliana*, tobacco and other model crops [67]. SH, a wild species closely related to AC, is named for the high density, large quantity, and abundant types of trichomes per unit area; compared with AC, it has many excellent agronomic traits, such as disease and pest resistance, cold tolerance and resistance to many other stresses, and is an important germplasm resource for modern tomato breeding [28, 68]. The trichomes of tomato are multicellular structures, the most studied being types I, IV, and VI. Types I and IV are the main sources of acyl sugars. Type VI glandular trichomes can produce large amounts of volatile monoterpenes, sesquiterpenes, phenylpropanoids, and flavonoids, which are plant volatile organic compounds that attract carnivorous predatory plant insects such as parasitic wasps and thus serve as an indirect defense against insects [69]. Cryo-SEM results showed that the different morphology and number of glandular cells in the rods and heads of the glandular trichomes and the different chemical substances secreted were the underlying reasons for the accumulation of a wider variety and quantity of secondary metabolites in SH than in AC, and were consistent with the results of the later metabolomic assay.

The results of the preference experiment showed that there was a significant difference in the resistance of AC and SH to aphids or mites after feeding for 1 h. Specifically, only a few aphids approached SH leaves and stayed briefly before moving towards AC leaves, and some aphids stayed around SH leaves for a long time before leaving. Furthermore, compared with aphids, mites were smaller, less susceptible to the external environment, and showed weak adaptability; after being placed in the center of the Petri dish, they made choices slowly, but also showed a tendency to prefer AC leaves.

Based on the above results, we speculated that when the plant is continuously fed or invaded by phytophagous insects, it may acquire resistance through secondary metabolites synthesized by trichomes. PSMs are the result of the interaction between plants and biological and abiotic factors in the environment during long-term evolution, and they can attract insects to help plants pollinate, or play an important role in plant defense against phytophagous insects as feeding and spawning inhibitors, and toxic substances. Identifying the key components and molecular regulatory mechanisms of insect resistance traits in SH and AC leaves has been the basis for insect resistance breeding and there is an urgent need to compensate for the small number of studies on this subject. Therefore, we determined the main components and content changes of trichome metabolism in AC and SH for the first time, and identified candidate differentially expressed metabolic pathways and key regulatory genes that regulate tomato resistance to insect invasion, in order to provide new strategies for enhancing tomato resistance.

With the development of sequencing technology, RNA-seq, as a routine experimental method for novel gene discovery, the study of quantitative gene expression, and transcript identification, provided the basis for identifying candidate genes for insect resistance and developing molecular markers [70]. In this study, annotation of all DEGs in the GO scheme identified the two terms with the most significant enrichment in biological process terms: defense response (GO:0006952) and response to adversity (GO:0006950). KEGG analysis indicated that the phenylalanine metabolism (ko00360) and monoterpene biosynthesis (ko00902) pathways were highly significantly enriched. It is worth noting that the phenylpropanoid biosynthesis pathway in plants starts from phenylalanine metabolism, and its products include lignin, flavonoids, and phenolic compounds, which play an extraordinary role in the secondary metabolic pathway of plants [71]. It can interact with other secondary metabolic pathways and work together with them to provide resistance to factors such as diseases, pests, cold, drought, UV, and nutrient stress during plant growth, at the same time, and it can also play an important role as a signal molecule and plant antitoxin in the interaction between plants and the external environment and microorganisms [72]. Therefore, the phenylpropanoid biosynthesis pathway was considered to be one of the important pathways affecting the synthesis of secondary metabolites and resistance in plants.

Moreover, *MYB* alone or in combination with *bHLH* and *WD40* proteins compose the *MBW* complex, which integrates target genes to regulate the synthesis of phenylpropanoids and flavonoids, due to the ability of *MYBs* to recognize and bind to the AC-rich elements in the promoters of *PAL*, *C3′H*, *4CL*, *CHS* and other genes [73–75]. To date, certain members of the *MYB* complex can activate dihydroflavonol 4-reductase (*DFR*), *bHLH*, *R2R3-MYB* repressor, and *R3-MYB* repressor [76]. Similarly, many *R2R3-MYB* activators of flavonoid biosynthesis have been characterized; James *et al*. [77] reported that physical interaction of poplar *MYB134* and *MYB115* with *bHLH131* led to over-accumulation of proanthocyanidins, which was induced by phytophagous insects, wounding, pathogen attack, UV-B exposure, and high light stress [78, 108]. The above results contributed to elucidating the important role of *MYB* transcription factors in the regulation of secondary metabolism.

Metabolomics, as an indispensable system biology tool, is the qualitative and quantitative analysis of small-molecule metabolite components of organisms under specific conditions, which leads to the analysis of the metabolic network of organisms with the aim of regulating the accumulation of secondary metabolites [79, 80]. Compared with other omics methods, metabolomics is an important component of systems biology, where metabolites are most closely related to phenotype [81, 82]. The use of metabolomics to analyze tomato fruit development, nutrient composition, and disease mechanisms is a current research hotspot, but the metabolic regulatory mechanisms of insect attack on tomato have been poorly studied. In this study, non-targeted metabolomics was used to detect 24 kinds of metabolites in positive ion mode, of which benzene and its derivatives accounted for 15%, and the proportion of benzene compounds in SH was higher than that in AC. Therefore, we speculated that SH may have strong phenylpropanoid metabolic activity, and the KEGG enrichment results were consistent with the results of transcriptome analysis.

Therefore, combining transcriptome and metabolome data, we determined that DEGs and DAMs were significantly enriched in the phenylpropanoid biosynthesis (ko00940) and flavone biosynthesis (ko00941) pathways. More specifically, ρ-coumaryl acid (ρ-CA) can be catalyzed by *PAL*, *C4H*, *4CL*, and *CAD* to synthesize ρ-coumaryl alcohol (C02646), thus providing precursors for different downstream branches of the metabolic pathway. *C4H* and *4CL* were co-expressed with *PAL* to complete the second and third steps of the phenylpropanoid metabolic pathway. Surprisingly, *4CL*, as a key branchpoint enzyme, connected the phenylpropanoid metabolism pathway and downstream metabolic pathways, while two *C4H*s and four *4CL*s were only identified in SH tomato and all were upregulated, which indicated that *C4H* and *4CL* were likely to be key regulatory genes for SH to resist insect invasion.

Compared with AC tomato, in SH the genes encoding *HCT*, *CAD*, *POD* enzymes were more higher expressed, which could thicken the secondary wall of SH leaves and promote the accumulation of 4-hydroxycinnamyl alcohol 4-d-glucoside (H-lignin), providing a good research basis for effective resistance to insect pest attack [39, 83]. Caffeoyl quinic acid (C00852), an important precursor of coniferyl alcohol and sinapyl alcohol, can be catalyzed by *COMT* to transform the above two metabolites, thereby promoting the accumulation of G- and S-lignin. Therefore, S-, G- and H-lignin were derived from three core lignin substrates: coniferyl alcohol, sinapyl alcohol, and ρ-coumaryl alcohol, respectively. Gesteiro *et al*. [84] found that the interdependence between ρ-coumaryl alcohol and lignin S monomers promoted resistance to stem-borer in maize cell-wall phenylpropanoids, which was consistent with the results of the present study. ρ-Coumaroyl CoA generated naringenin under the catalysis of *CHS* and *CHI* enzymes. Naringenin was a common intermediate that synthesized a variety of flavonoid subclasses, and can be catalyzed by *F3H* and *FLS* enzymes to accumulate kaempferol, thereby activating the metabolites related to the biosynthesis pathway of flavone and flavonol in SH to exert anti-insect effects [85]. Gómez *et al*. [86] analyzed the flavonoid components in soybeans by LC–MS and found that when the content of naringenin and kaempferol increased, this could effectively improve resistance against *Anticarsia gemmatalis* (Lepidoptera: Noctuidae). Flavonoids inhibited nematode invasion and hatching by affecting the motility and chemotaxis of *Meloidogyne incognita*, delayed insect resistance by affecting protease activity in some insects, and reduced the spread of insect pests and parasitic diseases by affecting the reproductive development of some insects. The above research results were consistent with the view from this study that SH tomato can inhibit pest invasion through the massive accumulation of naringenin and kaempferol.

Notably, Wang *et al*. [14] reported that *4CL*s were key regulatory points in the process of phenylpropanoid biosynthesis, and played important roles in plant development and defense. The present study was based on the specific upregulation of *Sl4CLL6* expression after 2 h of feeding by mites; it was hypothesized that *Sl4CLL6* may play a key role in regulating phenylpropanoid biosynthesis and responding to phytophagous insect attack. Subsequently, by silencing *Sl4CLL6* with 71.83% efficiency, it was found that when the *Sl4CLL6* gene was silenced the expression of downstream *SlHCT*, *SlCAD*, and *SlCHI* was also decreased, showing a cascade response of gene expression in specific biosynthesis pathways; this was consistent with the finding of Liu *et al*. [87] that knockdown of *GhOPR9* (12-oxo-phytodienoic acid reductase) also resulted in the reduction of *GhOPR3* expression in cotton.

Whole-plant infestation and the number of mites were used to analyze the ability of mites to reproduce the next generation on host plants and to spread on tomato plants, which was very important for assessing the destructive ability of the mites on host plants. In this study we observed infection symptoms and counted the number of mites on leaves of CK and silenced plants under the same conditions. Their propagation results showed that within 7 dpi of mite inoculation, mite populations increased on both CK and silenced plants, as evidenced by the fact that there was no significant difference in the number of mites on CK and silenced plants at 0 and 1 dpi, whereas the number of mites on silenced plants increased dramatically at 4 and 7 dpi, when it was 1.173- and 1.4-fold higher than that of CK, respectively, suggesting that the silencing of *Sl4CLL6* reduced resistance to mites in AC, leaving the leaves almost covered with mites. This was consistent with Li *et al*. [88], who reported that silencing of *Cm4CL2* altered *CmMYB15-like* regulation of key genes and metabolites of lignin biosynthesis and aphid feeding difficulties. In addition, the expression of *SlHCT* and *SlCHI* was changed in response to increased damage by mites. The above findings indicated that the phenylpropanoid metabolic pathway is a key pathway in response to insect pest stress, and when the key enzyme *4CL* in the phenylpropanoid metabolic pathway is knocked out using molecular biology, the metabolic flux of phenylpropanoid biosynthesis can be genetically controlled, which may lead to reprogramming studies of the metabolic pathways.

## Conclusion

Overall, the phenylpropanoid biosynthesis pathway is an important secondary metabolic pathway in plants, and SH can upregulate the expression of genes related to the phenylpropanoid biosynthesis pathway and be more resistant to phytophagous insects than AC, which will be of great value for further studies to evaluate the insect resistance function of *S. habrochaites*, and also to provide wild material resources for the genetic improvement of cultivated tomato.

## Materials and methods

### Plant material and insects

*Solanum habrochaites* (SH, LA1777, https://tgrc.ucdavis.edu/, National Plant Germplasm System (NPGS), CA, USA, accessed 15 October 2023) was supplied by the Tomato Genetics Research Center (University of California, Davis, CA, USA). ‘Ailsa Craig’ (AC) was harvested from the experimental base of the College of Agriculture, Northeast Agricultural University. Sterilized seeds were added to distilled water and placed in an oscillator until germination occurred. After germination, seeds were sown in plastic pots containing vermiculite and soil mixture (1:1 by volume) in an artificial climate chamber at 20–25°C with a photoperiod of 16 h/8 h (light/dark). When the plant reached the six-leaf stage, the second fully expanded leaf with the growth point down was randomly collected, a proportion of the total number of leaves was immediately frozen in liquid nitrogen and stored at −80°C until total RNA and metabolites were extracted; the other leaves was used for biotic stress treatment.

Aphids and *Tetranychus urticae* were provided by the College of Plant Protection, Northeastern Agricultural University. For each insect preference assay, a diameter was drawn on the bottom of 12 Petri dishes as a boundary and a circle with a diameter of 1 cm was drawn in the middle. At the left and right sides of the closed Petri dish were placed equal areas of AC and SH leaves, and the leaf types were marked on the dish walls. Ten mites were carefully transferred with a brush so as not to interfere with their motility. After covering the dishes with lids and observing for 1 h, we counted the number of insects that moved to the AC leaves or SH leaves and those that did not make a choice (NC), considering both sides of the boundary as preferential selection and around the boundary as not selected [89].

### Tomato trichome phenotypes imaged by cryo-SEM

Before observation, we poured liquid nitrogen into an open-topped dewar to fill the pipeline with high-purity nitrogen, cooling the SEM stage cold module to below −180°C. AC and SH leaves with different types of trichomes in the same stage of growth and development were rapidly freeze-fixed with liquid nitrogen slush, and then transferred to vacuum sputter for fracture under frozen conditions to expose the fresh fracture surfaces of the sample, and sublimation was followed by conductive spraying. After transferring the specimen holder to the SEM cold stage module, the voltage was set to 5 kV and the SEM sample stage position was adjusted for observation by rotating different control knobs [90].

### Sample preparation and extraction

Six biological replicates of each of the two tomato varieties, AC and SH, were taken for metabolomic analysis. Twelve samples were kept in a refrigerator at −80°C and were freeze-dried. Four hundred microliters of internal standard extract containing 70% methanol in water was added to 20 mg of sample and vortexing was performed for 3 min; after the samples were dispersed, they were taken out and placed in an ice bath, sonicated for 10 min and vortexed for 1 min, and then kept at −20°C for 30 min. Subsequently, the samples were centrifuged at 12 000 revolutions/min for 10 min at 4°C; 300 μl of the supernatant was centrifuged for another 3 min under the above conditions, and 200 μl was taken for metabolome analysis.

### UPLC–ESI–MS/MS conditions

All samples were acquired by the UPLC–MS (electrospray ionization, ESI) system following the machine’s instructions. UPLC conditions were as follows: a Waters ACQUITY UPLC HSS T3 C18 (1.8 μm, 2.1 mm × 100 mm) column was used; gradient elution was performed with the mobile phases of pure water containing 0.1% formic acid (A) and acetonitrile containing 0.1% formic acid (B), with injection volume 4 μl, flow rate 0.40 ml/min, and column temperature controlled at 40°C [91, 92]. MS–MS analysis was performed on an Agilent 6545 Q-TOF instrument equipped with an ESI turbo ion-spray interface to collect data in positive and negative ion modes, with ion-spray voltage +2500 V/−1500 V, Fragmentor 135 V, nebulizer 40 V, gas temperature 325°C, sheath temperature 325°C, and sheath flow 11 l/min [93].

### Identification of differentially accumulated metabolites and statistical analyses

The raw data detected by LC–MS were converted to mzXML format by ProteoWizard, and then the peaks were extracted, aligned, and corrected for retention time using the XCMS program. The peak areas were corrected by the SVR method, after which the peaks with a missing rate >50% were filtered out of each sample, and the metabolites were identified by using a database created by Frasergen Bioinformatics Co., Ltd (Wuhan, China). PCA, hierarchical clustering analysis (HCA), and OPLS-DA were preformed using R software to investigate the accumulation patterns of metabolites in AC and SH. Based on the OPLS-DA model, DAMs were screened with variable importance in projection (VIP) >1 and *P*-value <0.05. The KEGG Compound Database (http://www.kegg.jp/kegg/compound/) was utilized to annotate DAMs and map them to the KEGG Pathway Database (http://www.kegg.jp/kegg/pathway.html), which in turn identified significantly related metabolic pathways.

### RNA-seq and functional annotation

Three biological replicates each of AC and SH leaves were taken for BGISEQ RNA-seq by Frasergen Bioinformatics Co., Ltd (Wuhan, China) [94]. We evaluated the quality of total RNA after extraction. After the samples were proven qualified, library construction was carried out, and a single-stranded circular DNA library was obtained [95]. After the library construction was qualified, sequencing was performed according to the method described by Korostin *et al*. [96].

SOA Pnuke was used to finely filter the raw data to obtain valid clean reads and assess the quality of sequencing data. RNA-seq measured reads were quickly and accurately aligned to the AC genome using HISAT2 to remove redundancy and merge assemblies. Utilizing bowtie2, the second-generation sequence after quality control (QC) was aligned to the reference transcript sequence. Using RSEM, the comparison results of bowtie2 were called for statistics to get the number of reads compared with each transcript for each sample and converted to FPKM (fragments per kilobase million) to measure the differential expression of genes. Fold change (FC) between samples was determined using DESeq2 [97, 98]. The Benjamini–Hochberg method was chosen to complete the multiple hypothesis testing correction of *P*-values to obtain the FDR. DEGs were determined if |log_2_FC| ≥ 1 and FDR < 0.05. Volcano plots were constructed to show the distribution of all DEGs among samples. The functions of DEGs as well as pathway enrichment were annotated using KEGG, GO, and other databases.

### Correlation analysis of transcriptome and metabolome data

The correlation between DEGs and DAMs was calculated using Pearson’s correlation tests, and the PCC between genes and metabolites in the same pathway was obtained by running the cor package in R. Then, all metabolites and genes with coefficients ≥0.8 and *P*-value ≤0.05 were screened to construct heat maps, nine-quadrant diagrams, and correlation network diagrams, and to obtain the DEGs and DAMs that co-enriched the pathway information.

### Real-time RT–PCR analysis

From the transcriptome sequencing data, *4CL*, *CAD*, *HCT*, *PAL*, *UGT72E*, *F3H*, *CHS*, *CHI*, and *MYB78* were selected for qRT–PCR on two types of tomato leaves: fed on and not fed on by phytophagous insects. qRT–PCR was performed using 2 × SYBR Green qPCR Mix (with ROX) (Sparkjade, Shandong, China) and run on a CFX96 Touch Real-Time PCR System (Bio-Rad, CA, USA) for DEGs and transcription factors. Primer 5 (www.PremierBiosoft.com) was used to develop the primers listed in Supplementary Data Table S1. The amplification conditions and calculation of relative gene expression levels using the 2^−∆∆CT^ method were as described previously [99]. Selected untreated mites and mites fed for 2 h on AC and SH leaves were used for qRT–PCR analysis. For each sample we used three biological replicates and three technical replicates. The results are presented as the mean ± standard deviation. The primers used in this study are listed in Supplementary Data Table S1. Significance analysis and graphing were performed using Excel 2019, GraphPad Prism 5, SPSS 18.0, and other software.

### Multi-sequence alignment and phylogenetic classification

Phylogenetic trees were constructed using full protein sequences of *Arabidopsis thaliana* L., *Solanum tuberosum* L., *Oryza sativa* L., *Malus pumila* Mill., *Glycine max* L., and *Zea mays* L. and differentially expressed *4CL*s identified in AC and SH as a means of probing the evolutionary relationship of the *4CL* gene family. The full protein sequences are listed in Supplementary Dataset S2. Multiple sequence alignment and phylogenetic tree were performed using MEGA 5.0, neighbor-joining and bootstrapping methods were used to evaluate reliability, and the number of replications was 1000 [47].

### Effects of *Sl4CLL6* silencing on phenylpropanoid biosynthesis and effect on *Tetranychus urticae* resistance

Using the SGN VIGS Tool (https://vigs.solgenomics.net/) to intelligently select target sequences for silencing the *Sl4CLL6* gene [100], a 436-bp *Sl4CLL6* fragment was digested by XbaI and KpnI and ligated into the *pTRV2* vector. All recombinant vectors were transferred into *Agrobacterium tumefaciens* strain GV3101 and cultured and infiltrated as reported by He *et al*. [101] and Lian *et al*. [102]. Vacuum-infiltrated seedlings of AC were sown in substrate soil and then moved to an artificial climate chamber at 20–25°C with a photoperiod of 16 h/8 h (day/night) after 24 h of dark treatment [103, 104]. A total of 32 seedlings per treatment were replicated three times. Silencing efficiency in *pTRV2-Sl4CLL6* plants was assessed from obvious chlorosis and whitening symptoms in *pTRV2-PDS* plants [105].

After confirming the silencing of the *Sl4CLL6* gene, negative plants (CK) and silenced plants (*pTRV2-Sl4CLL6*) with consistent growth conditions were selected, and 20 adult mites were transferred at the same position to internodes 2 and 3 of the plants and each plant was placed separately in an incubator for cultivation. The level of resistance to *T. urticae* in AC was evaluated by observing infection symptoms and calculating the mean total number of live mites using a portable digital microscope on CK and *pTRV2-Sl4CLL6* plants at 0, 1, 4, and 7 dpi [106, 107]. The primers for VIGS vector construction of *Sl4CLL6* are listed in Supplementary Dataset S1.

## Acknowledgements

This work was supported by the National Natural Science Foundation of China (grants U22A20495 and 32072588) and the National Natural Science Foundation of Heilongjiang Province (LH2021C032).

## Author contributions

M.L.W. performed investigation, methodology and writing-original fraft. Y.D.W. performed formal analysis and visualization. X.Z.L. leveraged software. Y.Z., X.L.C., and J.Y.L. performed validation, supervision, and project administration. Y.W.Q. completed conceptualization, funding acquisition, acquisition of resources, and writing-review. A.X.W. performed project administration, acquisition of resources, and funding acquistion. All authors have discussed and approved the manuscript.

## Data availability statement

The authors declare that all data supporting the findings of this study are available within the article are available upon request from the corresponding author. The transcriptome raw data had been uploaded to the NCBI SRA database with the accession number PRJNA988827.

## Conflict of interests

The authors declare that they have no conflicts of interest.

## Supplementary information

Supplementary data is available at *Horticulture Research* online.

## Supplementary Material

Web_Material_uhad277
