# Supplementary material for: Integration of metabolomics and transcriptomics reveals the regulation mechanism of the phenylpropanoid biosynthesis pathway in insect resistance traits in Solanum habrochaites
Source: Hortic Res. 2024 Jan 9;11(2):uhad277. doi: 10.1093/hr/uhad277 (PMC10857935; doi:10.1093/hr/uhad277)
Supplement: Web_Material_uhad277 [file web_material_uhad277.zip › Dataset S1 Primers for VIGS vector construction of Sl4CLL6.doc]

Dataset S1 Primers for VIGS vector construction of *Sl4CLL6*

>S.lycopersicum ITAG4.0|Solyc08g076300.3.1 CDS

ATGGTAACATTCTTGAAAACCCAATTGAGCTCCCATGTCATAGAGGGTGAAGAAAGTGGGAATGGTGAAGTTGAGAAAAACCCTTCTAAAACCCATTCATTTCCCTTTTGGTACTGTGCTGAAACAGGTATATACAGCAGTAAGTACCCTTCCATAAAATTGCCAGAAGACCCTTTGCTTGATGTTGTTTCCTTCATTTTTTCTCACAAAAATGGTGGGGTTTCTGCTCTTGTGGACTCAGCTTCTGGCATTTCAATTTCCTATTCAGAGCTTTACCCCTTGGTTAAGTCTATGGCTAGTGGTCTTCAACAAAGGGGTGTTTCACAAGGTGATGTTGTTTTGATTCTACTCCCTAATTCCATTTATTTCCCTGTTATTTTGTTGGGTGTTTTGGCTCTTGGTGCAATTGCAACTACTATGAATCCCTTTAGCAATTTGTTAGAGATAAAGAAGCAAGCCCTTGATTGTTCTGTAACTTTGGCATTCACTTCTAGTGATAAAGTTGACCAATTGTCTACATTAGATATTCCAGTTATAGGGGTGCCAGAAATTTTGGTTTCTGGTTCGAATTGTAGTGAAAGTTCTGTTTTTTATGAGCTGATTTCCTGTGATCCTAATTGGGATTCAAGACCTAAAATAAGTCAGCAAGATACAGCGGCAATTCTGTACTCGTCTGGTACTACTGGTGTGGGAAAAGGCGTTATCTTGACTCATGGGAACTTTATAGCCATGGTGGAAACTTTTGTGAGGTTTGAAGCTTCACAATATGAATATTCGAGTTCTGAGAATGTGTATTTGGATGTTACACCAATGTTTCATGTGTATGGGCTTTCTCTCTTTGTGATGGGGTTGTTGTCGTTGGGAACTACCATTGTTGTGATGAGCAAATTTGACGCTGATGAGATGGTGAAAGCTATTGAGAGATACAATGTCACTCATTTTCCCCTAGTCCCACCATTACTAATGGCATTGACTAGAAGAGCAAAGGATGGTGCTTCGAGCAGTATGAAGAGCTTGAAACAGGTTTCATGTGGTGCAGCACCAGTAAACCCAAAATCCATTGAAGACTTCGTTCACACTCTCCCGGATGTTGATTTCATTCAGGGATATGGCATGACTGAGTCAACTGCTACTGGAACACGTGGTTACAATACTGAAAAGCTGCATAATTATTCTTCAGTAGGGCTTCTAGCTCCAAACATGCAAGCTAAAGTAGTGGATTGGATCACCGGTTCAACCTTAGCTCCCAACTGCATGGGTGAGCTCTGGCTTTGTGGACCTGGTGTAATGAAAGGATACTTAAATAACTTAGAGGCTACTAAGTCTACCATTGATGACAATGGTTATCTCCATACTGGTGATATTGCGTATTTTGATGAAGAAGGATATCTTTATGTCATTGACCGTCTGAAAGAGACTATCAAATACAAGGGATTTCAGATAGCTCCCGCTGATTTAGAATCTGTATTGGTCTCACATCCAGATATCATTGATGCTGCAGTAACAGGAGCCCGAGATGAGGAAGCTGGAGAAATTCCAGTAGCATTTGTCGTTAAGAGAGACGGGTGTGCAGTTTCCCAAACAGATGTAATAGACTTTGTTTCTAAGCAGGTTGCACCATTCAAGAGGATTAGAAAAGTTTACTTCAGAGCTTCCATACCCAGGTCTGCAGCTGGGAAGATACTTAGAAAGGAGTTAAGGAACATATTAACTTCCAAATTATAG

Synthesize amplification primers for *SL4CLL6*

Solyc08g076300.3.1(+): AAGGTTACCGAATTCTCTAGtgtgctgaaacaggtatatacagcagtaagtac
Solyc08g076300.3.1(-): GAGACGCGTGAGCTCGGTACaccttgtgaaacacccctttgttgaagac
