# Supplementary material for: Integration of metabolomics and transcriptomics reveals the regulation mechanism of the phenylpropanoid biosynthesis pathway in insect resistance traits in Solanum habrochaites
Source: Hortic Res. 2024 Jan 9;11(2):uhad277. doi: 10.1093/hr/uhad277 (PMC10857935; doi:10.1093/hr/uhad277)
Supplement: Web_Material_uhad277 [file web_material_uhad277.zip › Dataset S2 Phylogenetic analyses and multiple sequence alignments.doc]

Dataset S2 Phylogenetic Analyses and Multiple Sequence Alignments

>*At4CLL6*

MAATHLHIPPNPKTQTSHQNPPFWFSSKTGIYTSKFPSLHLPVDPNLDAVSALFSHKHHGDTALIDSLTGFSISHTELQIMVQSMAAGIYHVLGVRQGDVVSLVLPNSVYFPMIFLSLISLGAIVTTMNPSSSLGEIKKQVSECSVGLAFTSTENVEKLSSLGVSVISVSESYDFDSIRIENPKFYSIMKESFGFVPKPLIKQDDVAAIMYSSGTTGASKGVLLTHRNLIASMELFVRFEASQYEYPGSSNVYLAALPLCHIYGLSLFVMGLLSLGSTIVVMKRFDASDVVNVIERFKITHFPVVPPMLMALTKKAKGVCGEVFKSLKQVSSGAAPLSRKFIEDFLQTLPHVDLIQGYGMTESTAVGTRGFNSEKLSRYSSVGLLAPNMQAKVVDWSSGSFLPPGNRGELWIQGPGVMKGYLNNPKATQMSIVEDSWLRTGDIAYFDEDGYLFIVDRIKEIIKYKGFQIAPADLEAVLVSHPLIIDAAVTAAPNEECGEIPVAFVVRRQETTLSEEDVISYVASQVAPYRKVRKVVMVNSIPKSPTGKILRKELKRILTNSVSSRL

>*At4CL2*

MTTQDVIVNDQNDQKQCSNDVIFRSRLPDIYIPNHLPLHDYIFENISEFAAKPCLINGPTGEVYTYADVHVTSRKLAAGLHNLGVKQHDVVMILLPNSPEVVLTFLAASFIGAITTSANPFFTPAEISKQAKASAAKLIVTQSRYVDKIKNLQNDGVLIVTTDSDAIPENCLRFSELTQSEEPRVDSIPEKISPEDVVALPFSSGTTGLPKGVMLTHKGLVTSVAQQVDGENPNLYFNRDDVILCVLPMFHIYALNSIMLCSLRVGATILIMPKFEITLLLEQIQRCKVTVAMVVPPIVLAIAKSPETEKYDLSSVRMVKSGAAPLGKELEDAISAKFPNAKLGQGYGMTEAGPVLAMSLGFAKEPFPVKSGACGTVVRNAEMKILDPDTGDSLPRNKPGEICIRGNQIMKGYLNDPLATASTIDKDGWLHTGDVGFIDDDDELFIVDRLKELIKYKGFQVAPAELESLLIGHPEINDVAVVAMKEEDAGEVPVAFVVRSKDSNISEDEIKQFVSKQVVFYKRINKVFFTDSIPKAPSGKILRKDLRARLANGLMN

>*At4CL4*

MVLQQQTHFLTKKIDQEDEEEEPSHDFIFRSKLPDIFIPNHLPLTDYVFQRFSGDGDGDSSTTCIIDGATGRILTYADVQTNMRRIAAGIHRLGIRHGDVVMLLLPNSPEFALSFLAVAYLGAVSTTANPFYTQPEIAKQAKASAAKMIITKKCLVDKLTNLKNDGVLIVCLDDDGDNGVVSSSDDGCVSFTELTQADETELLKPKISPEDTVAMPYSSGTTGLPKGVMITHKGLVTSIAQKVDGENPNLNFTANDVILCFLPMFHIYALDALMLSAMRTGAALLIVPRFELNLVMELIQRYKVTVVPVAPPVVLAFIKSPETERYDLSSVRIMLSGAATLKKELEDAVRLKFPNAIFGQGYGMTESGTVAKSLAFAKNPFKTKSGACGTVIRNAEMKVVDTETGISLPRNKSGEICVRGHQLMKGYLNDPEATARTIDKDGWLHTGDIGFVDDDDEIFIVDRLKELIKFKGYQVAPAELEALLISHPSIDDAAVVAMKDEVADEVPVAFVARSQGSQLTEDDVKSYVNKQVVHYKRIKMVFFIEVIPKAVSGKILRKDLRAKLETMCSK

>*At4CL1*

MAPQEQAVSQVMEKQSNNNNSDVIFRSKLPDIYIPNHLSLHDYIFQNISEFATKPCLINGPTGHVYTYSDVHVISRQIAANFHKLGVNQNDVVMLLLPNCPEFVLSFLAASFRGATATAANPFFTPAEIAKQAKASNTKLIITEARYVDKIKPLQNDDGVVIVCIDDNESVPIPEGCLRFTELTQSTTEASEVIDSVEISPDDVVALPYSSGTTGLPKGVMLTHKGLVTSVAQQVDGENPNLYFHSDDVILCVLPMFHIYALNSIMLCGLRVGAAILIMPKFEINLLLELIQRCKVTVAPMVPPIVLAIAKSSETEKYDLSSIRVVKSGAAPLGKELEDAVNAKFPNAKLGQGYGMTEAGPVLAMSLGFAKEPFPVKSGACGTVVRNAEMKIVDPDTGDSLSRNQPGEICIRGHQIMKGYLNNPAATAETIDKDGWLHTGDIGLIDDDDELFIVDRLKELIKYKGFQVAPAELEALLIGHPDITDVAVVAMKEEAAGEVPVAFVVKSKDSELSEDDVKQFVSKQVVFYKRINKVFFTESIPKAPSGKILRKDLRAKLANGL

>*At4CL3*

MITAALHEPQIHKPTDTSVVSDDVLPHSPPTPRIFRSKLPDIDIPNHLPLHTYCFEKLSSVSDKPCLIVGSTGKSYTYGETHLICRRVASGLYKLGIRKGDVIMILLQNSAEFVFSFMGASMIGAVSTTANPFYTSQELYKQLKSSGAKLIITHSQYVDKLKNLGENLTLITTDEPTPENCLPFSTLITDDETNPFQETVDIGGDDAAALPFSSGTTGLPKGVVLTHKSLITSVAQQVDGDNPNLYLKSNDVILCVLPLFHIYSLNSVLLNSLRSGATVLLMHKFEIGALLDLIQRHRVTIAALVPPLVIALAKNPTVNSYDLSSVRFVLSGAAPLGKELQDSLRRRLPQAILGQGYGMTEAGPVLSMSLGFAKEPIPTKSGSCGTVVRNAELKVVHLETRLSLGYNQPGEICIRGQQIMKEYLNDPEATSATIDEEGWLHTGDIGYVDEDDEIFIVDRLKEVIKFKGFQVPPAELESLLINHHSIADAAVVPQNDEVAGEVPVAFVVRSNGNDITEEDVKEYVAKQVVFYKRLHKVFFVASIPKSPSGKILRKDLKAKLC

>*At4CLL5*

MASVNSRSGFCNSNSTFYSKRTPIPLPPNPSLDVTTFISSQAHRGRIAFIDASTGQNLTFTELWRAVESVADCLSEIGIRKGHVVLLLSPNSILFPVVCLSVMSLGAIITTTNPLNTSNEIAKQIKDSNPVLAFTTSQLLPKISAAAKKLPIVLMDEERVDSVGDVRRLVEMMKKEPSGNRVKERVDQDDTATLLYSSGTTGMSKGVISSHRNLIAMVQTIVNRFGSDDGEQRFICTVPMFHIYGLAAFATGLLAYGSTIIVLSKFEMHEMMSAIGKYQATSLPLVPPILVAMVNGADQIKAKYDLSSMHTVLCGGAPLSKEVTEGFAEKYPTVKILQGYGLTESTGIGASTDTVEESRRYGTAGKLSASMEGRIVDPVTGQILGPKQTGELWLKGPSIMKGYFSNEEATSSTLDSEGWLRTGDLCYIDEDGFIFVVDRLKELIKYKGYQVAPAELEALLLTHPEITDAAVIPFPDKEVGQFPMAYVVRKTGSSLSEKTIMEFVAKQVAPYKRIRKVAFVSSIPKNPSGKILRKDLIKIATSNSKL

>*At4CLL4*

MAYPERSLIVDPRSGFCKSNSTFYSKRQPLSLPPNLSRDVTTFISSQPHRGKTAFIDAATGQCLTFSDLWRAVDRVADCLYHEVGIRRGDVVLILSPNSIFIPVVCLSVMSLGAVFTTANTLNTSGEISKQIADSNPTLVFTTRQLAPKLPVAISVVLTDDEVYQELTSAIRVVGILSEMVKKEPSGQRVRDRVNQDDTAMMLYSSGTTGPSKGVISSHRNLTAHVARFISDNLKRDDIFICTVPMFHTYGLLTFAMGTVALGSTVVILRRFQLHDMMDAVEKHRATALALAPPVLVAMINDADLIKAKYDLSSLKTVRCGGAPLSKEVTEGFLEKYPTVDILQGYALTESNGGGAFTNSAEESRRYGTAGTLTSDVEARIVDPNTGRFMGINQTGELWLKGPSISKGYFKNQEATNETINLEGWLKTGDLCYIDEDGFLFVVDRLKELIKYKGYQVPPAELEALLITHPDILDAAVIPFPDKEAGQYPMAYVVRKHESNLSEKQVIDFISKQVAPYKKIRKVSFINSIPKTASGKTLRKDLIKLATSKL

>*At4CLL7*

MEKSGYGRDGIYRSLRPTLVLPKDPNTSLVSFLFRNSSSYPSKLAIADSDTGDSLTFSQLKSAVARLAHGFHRLGIRKNDVVLIFAPNSYQFPLCFLAVTAIGGVFTTANPLYTVNEVSKQIKDSNPKIIISVNQLFDKIKGFDLPVVLLGSKDTVEIPPGSNSKILSFDNVMELSEPVSEYPFVEIKQSDTAALLYSSGTTGTSKGVELTHGNFIAASLMVTMDQDLMGEYHGVFLCFLPMFHVFGLAVITYSQLQRGNALVSMARFELELVLKNIEKFRVTHLWVVPPVFLALSKQSIVKKFDLSSLKYIGSGAAPLGKDLMEECGRNIPNVLLMQGYGMTETCGIVSVEDPRLGKRNSGSAGMLAPGVEAQIVSVETGKSQPPNQQGEIWVRGPNMMKGYLNNPQATKETIDKKSWVHTGDLGYFNEDGNLYVVDRIKELIKYKGFQVAPAELEGLLVSHPDILDAVVIPFPDEEAGEVPIAFVVRSPNSSITEQDIQKFIAKQVAPYKRLRRVSFISLVPKSAAGKILRRELVQQVRSKM

>*At4CLL9*

MLTKTNDSRLIDRSSGFDQRTGIYHSLRPSLSLPPIDQPLSAAEFALSLLLKSSPPATAGKNIEALTYLVNSSSGDNLTYGELLRRVRSLAVSLRERFPSLASRNVAFILSPSSLDIPVLYLALMSIGVVVSPANPIGSESEVSHQVEVSEPVIAFATSQTVKKLQSSSLPLGTVLMDSTEFLSWLNRSDSSSVNPFQVQVNQSDPAAILFSSGTTGRVKGVLLTHRNLIASTAVSHQRTLQDPVNYDRVGLFSLPLFHVFGFMMMIRAISLGETLVLLGRFELEAMFKAVEKYKVTGMPVSPPLIVALVKSELTKKYDLRSLRSLGCGGAPLGKDIAERFKQKFPDVDIVQGYGLTESSGPAASTFGPEEMVKYGSVGRISENMEAKIVDPSTGESLPPGKTGELWLRGPVIMKGYVGNEKASAETVDKEGWLKTGDLCYFDSEDFLYIVDRLKELIKYKAYQVPPVELEQILHSNPDVIDAAVVPFPDEDAGEIPMAFIVRKPGSNLNEAQIIDFVAKQVTPYKKVRRVAFINAIPKNPAGKILRRELTKIAVDGNASKL

>*At4CLL1*

MESQKQEDNEYIFRSLYPSVPIPDKLTLPEFVLQGVEEYTENVAFVEAVTGKAVTYGDVVRDTKRLAKALTSLGLRKGQVMVVVLPNVAEYGIIALGIMSAGGVFSGANPTALVSEIKKQVEASGARGIITDATNYEKVKSLGLPVIVLGEEKIEGAVNWKDLLEAGDKCGDTDNEEILQTDLCALPFSSGTTGLQKGVMLTHRNLIANLCSTLFGVRSEMIGQIVTLGLIPFFHIYGIVGICCATMKNKGKVVAMSRYDLRIFLNALIAHEVSFAPIVPPIILNLVKNPIVDEFDLSKLKLQSVMTAAAPLAPELLTAFEAKFPNVQVQEAYGLTEHSCITLTHGDPEKGQGIAKRNSVGFILPNLEVKFIDPDTGRSLPKNTSGELCVRSQCVMQGYFMNKEETDKTIDEQGWLHTGDIGYIDDDGDIFIVDRIKELIKYKGFQVAPAELEAILLTHPSVEDVAVVPLPDEEAGEIPAACVVINPKATEKEEDILNFVAANVAHYKKVRAVHFVDSIPKSLSGKIMRRLLRDKILSINKK

>*At4CLL3*

MAYPERDLIVDPRSGFCKSNSTFYSKRNPLCLPPNPSLDVTTFISSQPQRGTTAFIDASTGHRLTFSDLWRVVDRVADCLYHEVGIRRGDVVLILSPNSIYIPVVCLSVMSLGAVVTTANTLNTSGEISKQIAQSNPTLVFTTSQLAPKLAAAISVVLTDEEDEKRVELTSGVRVVGILSEMMKKETSGQRVRDRVNQDDTAMMLYSSGTTGTSKGVISSHRNLTAYVAKYIDDKWKRDEIFVCTVPMFHSFGLLAFAMGSVASGSTVVILRRFGLDDMMQAVEKYKATILSLAPPVLVAMINGADQLKAKYDLTSLRKVRCGGAPLSKEVMDSFLEKYPTVNIFQGYALTESHGSGASTESVEESLKYGAVGLLSSGIEARIVDPDTGRVMGVNQPGELWLKGPSISKGYFGNEEATNETINLEGWLKLGDLCYIDEDGFLFVVDRLKELIKYKGYQVPPAELEALLIAHPHILDAAVIPFPDREAGQYPMAYVARKPESNLSEKEVIDFISNQVAPYKKIRKVAFISSIPKTASGKTLRKDLIKLSTSKL

>*At4CLL2*

MAVKHGVDGDGSEIESRTLAVDRKSGFCESTSIFYSKREPMALPPNQFLDVTSFIASQPHRGKTVFVDAVTGRRLSFPELWLGVERVAGCLYALGVRKGNVVIILSPNSILFPIVSLSVMSLGAIITTANPINTSDEISKQIGDSRPVLAFTTCKLVSKLAAASNFNLPVVLMDDYHVPSQSYGDRVKLVGRLETMIETEPSESRVKQRVNQDDTAALLYSSGTTGTSKGVMLSHRNLIALVQAYRARFGLEQRTICTIPMCHIFGFGGFATGLIALGWTIVVLPKFDMAKLLSAVETHRSSYLSLVPPIVVAMVNGANEINSKYDLSSLHTVVAGGAPLSREVTEKFVENYPKVKILQGYGLTESTAIAASMFNKEETKRYGASGLLAPNVEGKIVDPDTGRVLGVNQTGELWIRSPTVMKGYFKNKEATASTIDSEGWLKTGDLCYIDGDGFVFVVDRLKELIKCNGYQVAPAELEALLLAHPEIADAAVIPIPDMKAGQYPMAYIVRKVGSNLSESEIMGFVAKQVSPYKKIRKVTFLASIPKNPSGKILRRELTKLTTSKL

>Solyc02g088710.2.1

METHVVECSQKQEEDEHIFRSRYPPVHVPDNVTLPEFVLHNVELYADRLAFVDATTGKGYTYGEVERDIRRFAKALRSLGLRKGRVVLVVLPNVPEYAIVALGIMAAGGVFSGANPAAHSSEIMKQVESADGKLIVSDLPTYHKVKDCGLPVIILGEERAEGTIHWDELLEAADRASSRTDHITNQEDEIVQQSDLCALPFSSGTTGLSKGVMLTHRNLVANLCSTLFSISPEMIGQVTILGLIPFFHIYGITGICCATIRNKGKVVVMRRYELRAFLNALIIHEVSFAPIVPPIILALVKNPIVDEFDLKKLKLRSIMTAAAPLAPEILNEFEKKFPDVQVQEAYGMTEHSCITLSHSNQHIAKRNSVGFILPNLEVKFVDPDTGRSLPKNTPGEICVKSQCVMKGYYKNESETSLTIDTDGWLHTGDIGYIDDDGDIFLVDRIKELIKYKGFQVAPAELEGILLTHPSVEDTAVVGLPDEEAGEIPAAWVVLNSKAKESHEDIISYVASTVAQYKRVRLVQFVDSIPKSPSGKIMRRLIKEKMLERLKEGT*

>Solyc03g111170.2.1

MDPKNGYCGDTKIFKSLRPSVPLPPLTDPLSIVQYAFSLLNSPTTGINISATTFLIDADTGHCLSYADFLHQTQNLAASIQARYPSLSQNDVAFVLSPTSIHVPVVFFALLSLGVIVSPANPLSSSSELTHMVQLCKPLIAFATSSTVGKLPSSLFPLGTVLLDSPEFRKMIQIPISNPISYPIIHQSDSAAILYSSGTTGKVKGVELTHRNLIALIASLFYSRFNDDAANESEITEQAVAFMTLPLFHVFGFFMLIRLASMGEAMVITARFDFEKMLAAVEKYRVTYMPVSPPLVVAMAKSDLALKYDLSSLQLLACGGAPLGKEVAERFKSRFPNVEILQGYGLTETTGGATGMNGPEESNQYGSAGRLGVNVEAKIVDPESGKTLPPGQRGELWLRAPTIMKGYVGDKQATSATLDPEGWLKTGDLCYFDADGFLYVVDRLKELIKYKAYQVPPAELEHLLQSIPDVADAAVIPYPDEEAGEIPMAYIVRRPGSTISESQIIDIIAKQVSPYKKIRRVAFINAIPKSPAGKILRRELVNHATNGASARL*

>Solyc03g117870.2.1

MPMDTETNQSGDFIFRSKLPDIYIPKHLPLHSYCFENLSEFSSRPCLIDGSNDRIYTYAEVELTSRKVAVGLSKLGIQQKDTIMILLPNCPEFVFAFIGASYLGAISTMANPLFTPAEVVKQAKASSAKIVITQACFAGKVKDYAIENDLKVICVDSAPEGCVHFSELIQSDEHEIPEVNIQPNDVVALPYSSGTTGLPKGVMLTHKGLVTSVAQQVDGENANLYMHSDDVLMCVLPLFHIYSLNSVLLCALRVGAAILIMQKFDIAQFLELIQKHKVTIGPFVPPIVLAIAKSPLVDNYDLSSVRTVMSGAAPLGKELEDAVRAKFPNAKLGQGYGMTEAGPVLAMCLAFAKEPFDIKSGACGTVVRNAEMKIVDPDTGCSLPRNQPGEICIRGDQIMKGYLNDPEATARTIEKEGWLHTGDIGYIDDDDELFIVDRLKELIKYKGFQVAPAELEALLVNHPNISDAAVVPMKDEQAGEVPVAFVVRSNGSTITEDEVKDFISKQVIFYKRIKRVFFVETVPKSPSGKILRKDLRARLAAGISN*

>Solyc08g076300.2.1

MVTFLKTQLSSHVIEGEESGNGEVEKNPSKTHSFPFWYCAETGIYSSKYPSIKLPEDPLLDVVSFIFSHKNGGVSALVDSASGISISYSELYPLVKSMASGLQQRGVSQGDVVLILLPNSIYFPVILLGVLALGAIATTMNPFSNLLEIKKQALDCSVTLAFTSSDKVDQLSTLDIPVIGVPEILVSGSNCSESSVFYELISCDPNWDSRPKISQQDTAAILYSSGTTGVGKGVILTHGNFIAMVETFVRFEASQYEYSSSENVYLDVTPMFHVYGLSLFVMGLLSLGTTIVVMSKFDADEMVKAIERYNVTHFPLVPPLLMALTRRAKDGASSSMKSLKQVSCGAAPVNPKSIEDFVHTLPDVDFIQGYGMTESTATGTRGYNTEKLHNYSSVGLLAPNMQAKVVDWITGSTLAPNCMGELWLCGPGVMKGYLNNLEATKSTIDDNGYLHTGDIAYFDEEGYLYVIDRLKETIKYKGFQIAPADLESVLVSHPDIIDAAVTGARDEEAGEIPVAFVVKRDGCAVSQTDVIDFVSKQVAPFKRIRKVYFRASIPRSAAGKILRKELRNILTSKL*

>*Os4CL1*

MGSMEQQQPESAAPATEASPEIIFRSKLQDIAITNTLPLHRYCFERLPEVAARPCLIDGATGGVLTYADVDRLSRRLAAALRRAPLGLRRGGVVMSLLRNSPEFVLSFFAASRVGAAVTTANPMSTPHEIESQLAAAGATVVITESMAADKLPSHSHGALTVVLIDERRDGCLHFWDDLMSEDEASPLAGDEDDEKVFDPDDVVALPYSSGTTGLPKGVMLTHRSLSTSVAQQVDGENPNIGLHAGDVILCALPMFHIYSLNTIMMCGLRVGAAIVVMRRFDLAAMMDLVERHRVTIAPLVPPIVVAVAKSEAAAARDLSSVRMVLSGAAPMGKDIEDAFMAKLPGAVLGQGYGMTEAGPVLSMCLAFAKEPFKVKSGACGTVVRNAELKIIDPDTGKSLGRNLPGEICIRGQQIMKGYLNNPEATKNTIDAEGWLHTGDIGYVDDDDEIFIVDRLKEIIKYRGFQVAPAELEALLITHPSIADAAVVGKQIEPEIGEIPVAFVAKTEGSELSEDDVKQFVAKEVIYYKKIREVFFVDKIPKAPSGKILRKELRKQLQHLQQEA

>*Os4CL4*

MGSMAAAAEAAQEEETVVFRSKLPDIEIPSHLTLQAYCFEKLPEVAARPCLIDGQTGAVYSYGEVEELSRRAAAGLRRLGVGKGDVVMSLLRNCPEFAFTFLGAARLGAATTTANPFYTPHEIHRQASAAGARVIVTEACAVEKVRGFAADRGIPVVAVDGDFDGCVGFGEAMLDASIEPLDADEEVHPDDVVALPYSSGTTGLPKGVMLTHRSLVTSVAQQVDGENPNLYFRREDVVLCLLPLFHIYSLNSVLLAGLRAGSAIVIMRKFDLGALVDLTRRHGVTVAPFVPPIVVEIAKSPRVTADDLASIRMVMSGAAPMGKDLQDAFMAKIPNAVLGQGYGMTEAGPVLAMCLAFAKEPFEVKSGSCGTVVRNAELKIVDPDTGATLGRNQSGEICIRGEQIMKGYLNDPESTKNTIDKGGWLHTGDIGYVDDDDEIFIVDRLKEIIKYKGFQVPPAELEALLITHPDIKDAAVVPMIDEIAGEVPVAFIVRIEGSAISENEIKQFVAKEVVFYKRLNKVFFADSIPKSPSGKILRKDLRAKLAAGIPTNDNTQLKS

>*Os4CL2*

MITVAAPEAQPQVAAAVDEAPPEAVTVFRSKLPDIDIPSHLPLHEYCFARAAELPDAPCLIAAATGRTYTFAETRLLCRRAAAALHRLGVGHGDRVMVLLQNCVEFAVAFFAASFLGAVTTAANPFCTPQEIHKQFKASGVKLILTQSVYVDKLRQHEAFPRIDACTVGDDTLTVITIDDDEATPEGCLPFWDLIADADEGSVPEVAISPDDPVALPFSSGTTGLPKGVVLTHRSVVSGVAQQVDGENPNLHMGAGDVALCVLPLFHIFSLNSVLLCAVRAGAAVALMPRFEMGAMLGAIERWRVTVAAVVPPLVLALAKNPFVERHDLSSIRIVLSGAAPLGKELEDALRARLPQAIFGQGYGMTEAGPVLSMCPAFAKEPTPAKSGSCGTVVRNAELKVVDPDTGFSLGRNLPGEICIRGPQIMKGYLNDPEATAATIDVEGWLHTGDIGYVDDDDEVFIVDRVKELIKFKGFQVPPAELESLLIAHPSIADAAVVPQKDDVAGEVPVAFVVRAADSDITEESIKEFISKQVVFYKRLHKVHFIHAIPKSASGKILRRELRAKLAAC

>*Os4CL5*

MGSLPEQFVFRSRLPDIAIPDHLPLHDYVFERLADRRDRACLIDGATGETLSFGDVDALSRRVAAGLSSIGVCHGSTVMLLLPNSVEFAVAFLASSRLGAVTTTANPLHTPPEIAKQVAASGATVVVTEPAFVAKVSGLAGVTVVATGGGAERCASFAGLAAADGSALPEVAIDVANDAVALPYSSGTTGLPKGVMLSHRGLVTSVAQLVDGENPNLHLREDDVVLCVLPMFHVYSLHSILLCGMRAGAAIVVMKRFDTVKMLQLVERHGVTIAPLVPPIVVEMAKSDALDRHDLSSIRMVISGAAPMGKELQDIVHAKLPNAVLGQGYGMTEAGPVLSMCMAFAKEPTPVKSGACGTVVRNAELKIVDPDTGLSLPRNQPGEICIRGKQIMKGYLNNPEATEKTIDKDGWLHTGDIGFVDDDDEIFIVDRLKELIKYKGFQVAPAELEAMLIAHAAVADAAVVPMKDDSCGEIPVAFVVARDGSGITDDEIKQYVAKQVVFYKRLHKIFFVDAIPKAPSGKILRKDLRAKLAAGIPAC

>*Os4CL3*

MGSVAAEEVVVFRSKLPDIEIDNSMTLQEYCFARMAEVGARPCLIDGQTGESYTYAEVESASRRAAAGLRRMGVGKGDVVMSLLRNCPEFAFSFLGAARLGAATTTANPFYTPHEVHRQAEAAGARVIVTEACAVEKVREFAAERGVPVVTVDGAFDGCVEFREVLAAEELDADADVHPDDVVALPYSSGTTGLPKGVMLTHRSLITSVAQQVDGENPNLYFSKDDVILCLLPLFHIYSLNSVLLAGLRAGSTIVIMRKFDLGALVDLVRKHNITIAPFVPPIVVEIAKSPRVTAEDLASIRMVMSGAAPMGKDLQDAFMAKIPNAVLGQGYGMTEAGPVLAMCLAFAKEPFKVKSGSCGTVVRNAELKIVDPDTGTSLGRNQSGEICIRGEQIMKGYLNDPEATKNTIDEDGWLHTGDIGFVDDDDEIFIVDRLKEIIKYKGFQVPPAELEALLITHPEIKDAAVVSMKDDLAGEVPVAFIVRTEGSEITEDEIKKFVAKEVVFYKRINKVFFTDSIPKNPSGKILRKDLRARLAAGIPDAVAAAAADAPKSS

>*Os4CLL1*

MASASVPAAGYGADGVYRSLRPPAPVASDPGLSLTDLLLRRADACPSAVALADAAAGGRALTFAELRSAVLSTAVALSSRAGVRPGDAVLLLAPNCVLYPVCFFAVTALGAVGTTVNPDYTPREIAKQVSDARAKLVITISALVPKIAGLRLPVILLDDDANAAAASLPPDATVTLYTNLVAGVKEADYRRPPIKQSDTAALLYSSGTTGDSKGVILTHRNFIAAARMVTSDQDERREGPNVFLCFLPMFHIFGLSVITYAQLHRGNAIIAMSRFDINSLMEAVQRHRVTHLFCVPPVIIALAKHGKAGKYDLSSLKFIGSGAAPLGKDVMEVVAKKFPDSEIVQGYGMTETCGIISLEYPEKGQAREFGSTGTLVSGVEAKIVDIKTLKHLPPNQVGEICVRGPNVMQGYFNNVQATEFTIKQGWLHTGDLGYFDGGGQLFVVDRLKELIKYKGFQIAPAELEGLLLSHPEILDAVVIPFPDAKAGEVPIAYVVRSPDSSLTEVDVQKFIEKQVAYYKRLKRVTFVGSVPKSASGKILRRQLIAQVRSSKL

>*Os4CLL7*

MAAPTSMANADGESTRTGYCAATKSFRSLRPPVPLPPPDVPLSFPEFAFSLLPRSSSSSSSSLLPANPALVDAATGEAVSFQAFLSRVRALAGALRSRVGLRGGDVAFVLAPAGLDVPVLYFALLSIGAVVSPANPALTPAEVSRLVSLSGASVAFAVSSTATKLPAGLTTVVLLDSPHFRSLLMDCGQAQGQEPLPVVVVRQSETAAIQYSSGTTGRVKAAALPHRSFIAMVAGFHALRAKAREVRTLLGAPMFHSMGFLFVLQGVALGATTVVVTDAVARAGIRGLVEAAERWAVMDMTASPPVVLGMTKQRCRLPALERITCGGAPLPAPAIERFRRRFPHVDLCMGYGSTEAGGISRMISQEECNHIGSAGRVTENVEVKIVDHVTGKPLPAGQQGELWVRGPAVMTGYVGDNEANATTFNSEGWLKTGDLCYIDQDGFLFVVDRLKELIKYKAYQVPPAELELVLHSLPQIVDAAVMPYPHEEAGQIPVALVVKQPGSKLTEAEVMYNVAKQVAPYKKIRKVLFVDSIPKSPSGKILRRELVNHLRLCELSRL

>*Os4CLL2*

MQPDAAATAHPSLSFYSAATGLYSSLHPPLPLPSDPSLSLVPHLFSHLPLHHHSLLVDAPTAATLSCADFRRLVSSLAAGLRRRLHIARGSLVLLLLPNSLTFPVAFLAVLATGAVATTMNPSSAPAEIAARLRDTAPSLVLASTHNAAKLPPLAAPLVLVPDTFQQQHDDDQFDFFFHALLETDPETPVEMGVGVGQDDAAAVLYSSGTSGRSKGVVVTHRNLIAMVELFVRFEASQYTRPARDNVYLAALPMFHVYGLSLFAVGLLSLGCTVVVMRRFNVDDAVKAIRKYKVTHLPLVPPIMSALLRANPPLELDSLLQVSSGAAPLNHTLIHHFLHAFPHVDFIQGYGMTESTAVGTRGFNTCKHKKYASVGLLAPNMHAKIVHLESGSCLPPGSYGELWLHGPAIMKEFCFVTGYLNDDDDAFTRKDGWLRTGDIAYFDSDGYLFIVGRLKDTIKYKGFQIAPADLEAVLIRHPEIIDVAVTSDEDEEAGEIPVAFVVRKSGSTLSCTHVMEYVAKQVRTRRSYTTQHSPYFLAWEHYKIIITASAQSAFLLARVSNSSKNGSLQALCPPVQINMADIS

>*Os4CLL6* MSKRSPPPQPAHIPMAEQRWRPPYPYASSAAGGGGVDRRSRSGFCAATRTFHSLRSVGPLPPEELPLTVAAYAFSLLSSAPPLVVAGRGPALVDAATGIAVSYPAFVARVRFLAGGLWCSLGLRPGDVALVVSPSCLDVAVLYFALMSIGVVVSPANPASTADEYAHQVRLSRPAIAFVAPEVAARLPRHVSRVVIGSEVFDRLASASAAGGWAAPPAVAMKQPSTAALLYSSGTTGRVKAVAITHRNLIAQISAYNAIRETVAREAATDAGKGKPPPPSPSPPAAVTLFPLPLFHVMGFGLLTRTISSGETAVVMRRFDLAAAARAVERYRVTKLSAAPPVVVALTKSDEARRRDLSSLVAIVVGGAPLGREVSQRFATVFPSVQIVQSYGLTESTGPVATMAGPEESAAYGSVGRLAPRVQAKIVDTATGEVLGPGRRGELWIRGPVVMKGYVGDPEATAATITPDGWLKTGDLCYFNEDGYLYVVDRLKELIKYKGYQVPPAELEHILQSRPEIADAAVVPYPDEEAGQLPMAFVVRQPGAYLTEQQVMNCVAKHVAPYKKVRRVAFVNAIPKSPAGKILRRELVLQAMASTSRL

>*Os4CLL4*

MGRSPEMEVDARSGYCAATRTFRSRRADVPLPADPEVDVVSFLASRRHSGVVALVDAATGRRITFTELWRAVAGAASALAAHPVSLRKGHVALILSPNSVHFPVAALAAMSLGAVLTTANPLNTPAEIAKQVADARPVLAFTTRELLPKLPRAHDLRVVLLESARLPGDSSDPRIVATIEEISATTPDPARRKDRVTQDDPATLLYSSGTTGPSKGVVATHRSLISMVQIIMTRFRLEGSDKTETFLCTVPMFHVYGLVAFATGLLGCGATVVVLSKYELPEMLRSINAYGVTYLPLVPPILVAMVAHPKPLPLGQMRKVLSGGAPLGKELIEGFREKYPQVEILQGYGLTESTAIGASTDSAEESRRYGTAGLLSPNTEAKIVDPDSGEALPVNRTGELWIRGPYVMKGYFKNAEATQSTLTPDGWLKTGDLCYIDEDGYLFVVDRLKELIKYKGYQVPPAELEALLLTHPEVTDVAVIPFPDREVGQFPMAYIVRKKGSNLSEREVMEFVAKQVAPYKKVRKVAFVTDIPKNASGKILRKDLIKLATSKL

>*Os4CLL8*

METELHLAAGYCAATGVYRSGHPPQFAAAAALSFPEYILPHMLLPGRRARPAFVDASTGAALSFAGLRALSLRVARALAAAGLRRGRVALLLSPNSLHFPALSLAVLSLGAVLSAANPLLTPDELARQADDAKPFLALVTGELAPKLRSIAPDVKLVLVEQLLADVAAEVDDDETLDLPAANIGRDDAALLFYSSGTTGRSKGVVSTHGNAIAMAASLERAWGGGGGGGEKPQQYDDHDEAYGCVLPMFHMFGFSSFVMGTAALGATAVVVPGRFSVEKTMAAVEEYGVTRLLVVPPMVVKMVAAAAGDGEPSRRRLRLRQVVSSGAPLQREHMARFRSCFPAVNLGQCYGLTETTGIVTMCDLQHNDNGIDKVEMPPSSTDMTFVAVAATTTEVKERSTGGGGGGGGVSIGRLMPDVEAKIVDPDSGELLPPRRTGELWVRGPSTMRGYLNNEEATALALVAAAGSVSVSGGGERWLRTGDLCYVDSRGLVYVVDRVKELIKCNAYQVAPAELEDVLATHPDIHDAAVAPYPDKEAGEIPMAYVVKKQGSGHLQEDEVISFVQNKVAPYKKIRKVVFVDSIPRSPSGKILRRQLKNLLQGSILHRSRM

>*Os4CLL5*

MADRAPPPPPPPAGIDSRSGFCAATRIFHSTRAPGDLPPESLPMTAAAYAFSLLSSSTLPGRPALVDAATGIAISYPSFLAAVRSLAGGLWCSLGLRPGDVALVVAPSRLEVPVLDFALMSIGAVVSPANPVSTPEEYAHQVALSRPVVAFAAPEVAAKLPEHVRCVVIGSDEYGRLAASDGRRAAAPAAVAVKQSDTAAVLYSSGTTGRVKAVAITHRNLIALMSLHADNREKVAREAAEAGEEPPPPAVTLLPIPLFHVFGFMMVLRSVSMGETSVLMERFDFIAALRAIERYRVTLLPAAPPVLVAMVKYEEARRRDLSSLLVIGIGGAPLGREVAEQFASVFPNVELVQGYGLTESSGAVAATVGPEESKAYGSVGKLGSHLQAKIVDPSTGYVGDDEATAATVDSEGWLKTGDLCYFNEDGFLYIVDRLKELIKYKGYQVPPAELEHILQSHPGIADAAVIPYPDEEAGELPMAFIVRQPGSNITKEQVMDYVAKQVAPYKKVRRVAFVTAIPKSPAGKILRRELVQQALSMGASKL

>*Os4CLL3*

MQRDAIAAARNAGCSSGRISQPPPPPFYSAATGIYSSIHPPVALPTDPSLTLVAHLFARLPLADPGAPTLVDAATASAVSRADLRRLVASLAAGLRRRHGVRKGSVVLLLLPNSVAFPVSFLAVLAAGAVATTMNPSSSPAEIAAQARATGACLVLASRDGAARLPPLAAPVVLVPEILDHSAAADDGDDDQRVFAAFRAMLDGGGGDGTETAVPVVGQDDAVAILYSSGTSGRSKGVVLTHRNLIAMTELFVRFEASQYHARGARENVYMAALPMSHVYGLSLFAVGLLSIGATVVVMRRFDAGDAVAAIGRYKVTHMPLVPPIMAAMVRAAAAGGVPPSQVASLVQVSCGAAPITAALIHEFLQAFPHVDFIQGYGMTESTAVGTRGFNTSKHKKYTSVGLLAPNMHAKIVHLESSSCLPPGFSGELWLHGPGIMKGYLSDDDDACTRKDGWLRTGDIAYFDLDGYLYIVGRLKDTIKYKGFQIAPGDLEEVLIHHPEILDVAVTSAEDEEAGEIPVAFVVRRSGSNLSCKQVMEYVAKQVAPYKRVRKVVFVEAIPKSPAGKVLRRLLRNSHDTAAAATSSCSISSKL

>*Os4CLL9*

MGDAAVPAMVVEEEEQEHVFRSRFPPVAVPDGVTVPEFVLDGAEAYADRVALVEAAAGGRSYTYGEVARDTARFARALRSVGVRKGHVVVVALPNLAVYPVVSLGIMSAGAVFSGVNPRALAAEIKKQVEDSEAKLVVANEVAFDKVKDAGVPVIGVGDRERMPGAISWDGLLAAADRTGAGVVPVDAAQQSDLCALPYSSGTTGVSKGVMLSHRNLVSNLCSSMFAVAPETAGQVVTLGLMPFFHIYGITGICCATLRHKGTVVVMDRFDLRTFLRALVDHRVMFAPLVPPVMLAMVKSPVADEFDLSDLALKSVMTAAAPLAPDLLAAFQRKFPGVQVEEAYGLTEHSCITLTHAAGDGHGHVAKKSSVGFILPNLEVKFVDPDTGRSLPANTPGELCVRSQSVMQGYYKRKEETERTVDGKGWLHTGDVGYIDGDGDVFIVDRIKELIKYKGFQVAPAELEAVLLSHPSVEDAAVFGVPDEEAGEVPVACVVRRHGAEEGEEEIVAYVAERVASYKRVRVLHIVDAIPKSVSGKILRRQLRDEFIKRMKPSA

>*Gm4CL2* MITLAPSLDTPKTDQNQVSDPQTSHVFKSKLPDIPISNHLPLHSYCFQNLSQFAHRPCLIVGPASKTFTYADTHLISSKIAAGLSNLGILKGDVVMILLQNSADFVFSFLAISMIGAVATTANPFYTAPEIFKQFTVSKAKLIITQAMYVDKLRNHDGAKLGEDFKVVTVDDPPENCLHFSVLSEANESDVPEVEIHPDDAVAMPFSSGTTGLPKGVILTHKSLTTSVAQQVDGENPNLYLTTEDVLLCVLPLFHIFSLNSVLLCALRAGSAVLLMQKFEIGTLLELIQRHRVSVAMVVPPLVLALAKNPMVADFDLSSIRLVLSGAAPLGKELEEALRNRMPQAVLGQGYGMTEAGPVLSMCLGFAKQPFQTKSGSCGTVVRNAELKVVDPETGRSLGYNQPGEICIRGQQIMKGYLNDEAATASTIDSEGWLHTGDVGYVDDDDEIFIVDRVKELIKYKGFQVPPAELEGLLVSHPSIADAAVVPQKDVAAGEVPVAFVVRSNGFDLTEEAVKEFIAKQVVFYKRLHKVYFVHAIPKSPSGKILRKDLRAKLETAATQTP

>*Gm4CL1* AKATILLMPKFDINSLLALIHKHKVTIAPVVPPIVLAISKSPDLHKYDLSSIRVLKSGGAPLGKELEDTLRAKFPNAKLGQGYGMTEAGPVLTMSLAFAKEPIDVKPGACGTVVRNAEMKIVDPETGHSLPRNQSGEICIRGDQIMKGYLNDGEATERTIDKDGWLHTGDIGYIDDDDELFIVDRLKELIKYKGFQVAPAELEALLLTHPKISDAAVVPMKDEAAGEVPVAFVVISNGYTDTTEDEIKQFISKQVVFYKRINRVFFIDAIPKSPSGKILRKDLRAKIAASVPK

>*Gm4CLL7L*

MQRSGYGSDGIYRSLRPSIVFPKNSNLSLVSHLFNRVAAFPSKPALIDADSSETLSFAELKLLTVRVAHGLLRLGVTKNDVVLFLAPNDIRYIVCFLAVASLGAAVTTVNPAYTAAEVSKQANDSKPKLLVTVAELWDKLEHLKLPAVFLRCSNAPHAPSSATSFDALVQLAGSVTEFPEIKIKQSDTAALLYSSGTTGLSKGVVLTHGNFVAASLMIGFDDDLAGVLHSVFLCVLPMFHVFGLMVISYGQLQRGSAVVSLKKFEFELVLKTIEKFKVTHLWVVPPIILALAKHGLVDKYDLSSLKHIGSGAAPLGKELMKECAKRFPHAIVSQGYGMTETCGIVSVENARMGIRNSGSTGMLVAGMEAQVVSVDTLKPLPPGQLGEIWVRGPNMMQGYHNNPQATRLTMDKKGWVHTGDLGYFDEDGQLFVVDRIKELIKYKGFQVAPAELEGLLVSHAEILDAVVIPYPDAEAGEVPVAYVVRSPNSSLTEEDVQKFIAKQVAPFKRIRRVTFINAVPKTASGKILRRELIEKVRSKI

>*Gm4CL4*

MITLAPSLDTPKTDQNQVSDPQTSHVFKSKLPDIPISNHLPLHSYCFQNLSQFAHRPCLIVGPASKTFTYADTHLISSKIAAGLSNLGILKGDVVMILLQNSADFVFSFLAISMIGAVATTANPFYTAPEIFKQFTVSKAKLIITQAMYVDKLRNHDGAKLGEDFKVVTVDDPPENCLHFSVLSEANESDVPEVEIHPDDAVAMPFSSGTTGLPKGVILTHKSLTTSVAQQVDGENPNLYLTTEDVLLCVLPLFHIFSLNSVLLCALRAGSAVLLMQKFEIGTLLELIQRHRVSVAMVVPPLVLALAKNPMVADFDLSSIRLVLSGAAPLGKELEEALRNRMPQAVLGQGYGMTEAGPVLSMCLGFAKQPFQTKSGSCGTVVRNAELKVVDPETGRSLGYNQPGEICIRGQQIMKGYLNDEAATASTIDSEGWLHTGDVGYVDDDDEIFIVDRVKELIKYKGFQVPPAELEGLLVSHPSIADAAVVPQKDVAAGEVPVAFVVRSNGFDLTEEAVKEFIAKQVVFYKRLHKVYFVHAIPKSPSGKILRKDLRAKLETAATQTP

>*Gm4CLL5*

MSGEEILSSNEEKYNRSQPPFGSRSGYNSRTGIYHSLIKLGIKHEIPTRPDLNTANFVLSQFPQAHLAEARIAFIDARTNLSVSYGELRRSIYSLASALFHGLEVRKGDVVFVLSPNSTLYSTICLAVLSVGAVLTTANPINTATEIAKQVHDSSAKLAISAPEELHKLVPTGVPTILTSHCSDGNMLSVEELIKGCCTSPELPQVPVAQSDTAAILYSSGTTGVSKGVVLTHANLISIMRLLLWSADVSGSQDDVFLAFIPMFHIYGLVFFGLGLLCVGVTTILMQKYDFQAMLDAIQKHKVNNLPAVPPVILALVKHARKARCDLSSLRRVGSGAAPLSKEVALEFRRMFPWIELRQGYGLTESSGGATFFASDKDAKAHPDSCGKLIPTFCAKVVDIEKGKPLPPHKEGELWFKSPTIMKGYLGNLEATSAAIDSEGWLRTGDLGYIDENGFVYIVERIKELIKHNGYQVAPAELESVLLSHPLIVDAAVIPVEDEETGQIPMAYVVRAAGSELSENQVIQFVAGQVAPYKKVRKVSFIDTIPKSAAGKILRKDLVSQSKYQLVSKLRVSIFQWIFNKIMSRKEERSSRKDEYNKSLPPFDTRSGYDSRTGIYHSLVKLGTKHEIPTKPDLGTANFVLSQFPQAHLAEARIAFIDSGTNRSVSYGELRRSIYSLASALFNRLKVRKGDVVFVLSPNSTLYSTICLAVLSVGAVVTTANPINTESEIAKQVHDSGAKLAISTLEDLHKLVPTGIPTILTSRPFDGNMLSIEELIEDCYGSPQLPQVPVAQSDTAAILYSSGTTGRSKGVLLTHANIISIMRLLFWQVDVSGSQDDVFFAFIPMFHIYGMIFFGLGLLCIGITTVLMQKYDFQAMLVAIQKYKVNNLPAVPPVILALVKHSSKVKCDLSSLKRVGSGAAPLSKEVAQEFRRMFPSVELRQGYGLTESSGGAAFFASDKDAKAHPDSCGKLIPTFCAKVIDIETGKPLPPRKEGELWFKSPTIMKEYLGNMEETSATIDSEGWLRTGDLGYIDENGFVYIVERIKELIKHNGYQVAPAELESVLLSHPLIVDAAVIPVEDEETGQIPMAYVVIAAGSELSEDQVIQFVAGEVAPYKKVRRVSFIDTIPKSAAGKILRKDLVSQSRHQLVSKL

>*Gm4CLL9*

MEQRKSIWSPVTDPRSGFNRESRTFHSLKPPLRLPPPNAAVSAAAFALFLRRNSQFPDSSTAFIDSATGHRLSYGELLHRAKTLASNLATILKLTKGDTALVLSPNILQVPILCFALLSLGVVVSPANPLSTRSELTRFFNISNPSIVFTVTSVVEKTREFQVKTVLLDSPEFDTLTKSQIHTKYIQECDSHQRGLVSNMQVTQSDVAAILYSSGTTGTIKGVMLTHRNLTAIAAGYDTVREKRKEPAVVLYTVPFFHVYGFTFSLGAMVLSETVVIMERFSMKAMLSAVERFRVTHATMVPALVVAMTKDCVIAGYDLTSLEGIVCGGSPLRKETDEAFKAKFPNVLVMQGYGLTESAVTRTTPEEANQVGATGKLIPNIEAKIVNPETGEAMFPGEQGELWIRGPYVMKGYSGDPKATSATLVDGWLRTGDLCYFDSKGFLYVVDRLKELIKYKGYQVAPAELEELLLSHSEINDAAVIPYPDEVAGQVPMAFVVRQPQSSLGAAEVIDFVAKQVSPYKKIRRVAFVNSIPKNAAGKILRKDLKLALSRL

>*Gm4CLL7*

MQKAGYGGDGIYRSLRPCLVLPNDPNLSLVSFLFQSVSSFPSKIALVDSHSSQTLTLAHLKSQVAKLAHGFLKLGINKNDVVLLLAPNSIHYPICFLAATAIGAVVSTANPIYTVNEISKQVDDSNPKLLITVPELWDKVKNLNLPAVIIDTETAQGLVSFEAGNEVSRITSLDAVMEMAGPATELPESGVKQGDTAALLYSSGTTGLSKGVVLTHRNFIAASVMIGMDDDLAGEQDDVYLCVLPMFHVFGLAVVTYAALRRGSAVVVMERFELEALLKAIEKQRVTKLWVVPPILLGLAKQSVVGNYDLSSLRRIGSGAAPLGKDLMEECGRRFPHVAICQGYGMTETCGIVSVENPRVGVRHTGSTGTLVSGVEAQIVSVDTQKPLPPRQLGEIWVRGPNMMQGYHNNPEATRLTIDKKGWVHTGDLGYFDEDGQLYVVDRIKELIKYKGFQVAPAELEGLLVSHPEILEAVVVPNSHLSYFLCNRYPDDEAGEVPIAYVVRSPNSSLTEEEIQKFIAKQVAPFKKLRRVTFINNVPKTASGKILRRELTAKARSKI

>*Zm4CL2*

MGDAAIAVVHEADEEEQEHIFRSRFPPVDLPDDVTVPEFVLAGAEAYADKVALVEAAPGGRSYTYGEVARDVARFARALRSVGVRKGHVVVVALPNLAVYPVVSLGIMSAGAVFSGVNPRAIAAEIKKQVEDSDAKLVVTNEVAYDKVKDAGVPVIGIGDDMERLPGAISWDELLAAADRTGAPVVALDPVQQSDLCALPYSSGTTGVSKGVMLSHRNLVSNLCSSMFAVGEELVGQVVTLGLMPFFHIYGITGICCSTLRHKGTVVVMDRFDLRTFLGALVTHRVMFAPVVPPVMLAMVKSPVADDFDLSGLALRSIMTAAAPLAPDLLAAFEKKFPGVQVEEAYGLTEHSCITLTHAASGDDARQGPVQVAKKNSVGFILPNLEVKFVDPDTGRSLPKNTPGEICVRSQAVMQGYYRKKEETERTIDAKGWLHTGDVGYIDDDGDVFIVDRIKELIKYKGFQVAPAELEAILLSHPSVQDAAVFGLPDEEAGEIPVSCVVRRCGASESEADIMAYVAGRVASYKKLRLLQFVDVIHKSVSGKILRRQLRDEFVSRTKTAAA

>*Zm4CL1*

MGSVDAAIAVPVPAAEEKAVEEKAVVFRSKLPDIEIDSSMALHTYCFGKMGEVAERACLVDGLTGASYTYAEVESLSRRAASGLRAMGVGKGDVVMSLLRNCPEFAFTFLGAARLGAATTTANPFYTPHEVHRQAEAAGARLIVTEACAVEKVREFAAERGIPVVTVDGRFDGCVEFAELIAAEELEADADIHPDDVVALPYSSGTTGLPKGVMLTHRSLITSVAQQVDGENPNLYFRKDDVVLCLLPLFHIYSLNSVLLAGLRAGSTIVIMRKFDLGALVDLVRRYVITIAPFVPPIVVEIAKSPRVTAGDLASIRMVMSGAAPMGKELQDAFMAKIPNAVLGQVRPSSSSEAFSVFLMTRVPFRSHSDQCNAMQQGYGMTEAGPVLAMCLAFAKEPYPVKSGSCGTVVRNAELKIVDPDTGAALGRNQPGEICIRGEQIMKGYLNDPESTKNTIDKDGWLHTGDIGYVDDDDEIFIVDRLKEIIKYKGFQVPPAELEALLITHPEIKDAAVVSMNDDLAGEIPVAFIVRTEGSQVTEDEIKQFVAKEVVFYKKIHKVFFTESIPKNPSGKILRKDLRARLAAGVH

>*Zm4CLL9*

MGDAAIAAVHLHESEEEHIFRSRFPPVAVPDDVTVPEFVLADAEAYADKTALVEAAPGGRSYTYGELVRDVARFARALRSIGVRRGHVVVVALPNLAVYPVVSLGIMSAGAVFSGVNPRAVAAEIKKQVEDSEARLVVADAVAYDKVKDAGVPVIGIGDVARLPGAIGWDELLAMADRAGAPVVALEPAQQSDLCALPYSSGTTGVSKGVMLSHRNLVSSLCSSMFAVGQELVGQVVTLGLMPFFHIYGITGICCATLRHKGTVVVMDRFDLRAFLGALLTHRVMFAPVVPPVMLAMVKSPVADEFDLSGLALRSVMTAAAPLAPDLLAAFERKFPGVQVEEAYGLTEHSCITLTHASGGGEDVGSAVQVAKKKSVGFILPNLEVKFVDPDTGRSLPKNTPGEICVRSQAVMQGYYRKKEETERTIDAAGWLHTGDVGYIDDDGDVFIVDRIKELIKYKGFQVAPAELEAILLSHPSVEDAAVFGLPDEEAGEVPASCVVRRRGAPESEADMMAYVAGRVASYKKLRLLRFVDAIPKSVSGKILRRQLRDEFVKKTAAA

>*Zm4CLL1*

MVGTVSLAAGYGSDGVYRSPRLVAAIASDPGLSVTDLVLRRAAACPSAPALVDAATGRALTFGALRSAVLVAAAALSSRAGVRRGDAVLLLAPNCVLYPVCFLAVTALGAVATTANPLYTPREIAKQAADARARLVVTVSDLLPKISGLRLPIVLLDGDGAPVPSGCPSSNVTLYSDLVAGVQEAEYRHTPIGQGDTAALFYSSGTTGESKGVVLTHRNFIAAATMVTSDQDELGEGHNVFLCFLPMFHIFGMSVITLGQLQRGNAVVVMARFDVDAVLAAIERHRVTYLFCAPPAMIALAKHSRGGRYDLSSLRCIGSGAAPLGKDVMVAMADRFPGVDIIQGYGMTETCGIISLEYVQKGCARQFGSTGALVTGVEAKIVNAKTMKHLPPSQLGEICVRGPNIMEGYFNNVQATESTIKNGWLHTGDLGYFDERGQLHVVDRLKELIKYKGFQIAPAELEGLLLSHPEILDAAVIPYPDAEAGEVPIAYVVLSPKSSLAEADVQKFIEKQVAHYKRLRKVTFVGSVPKSAAGKILRRELIAQVRLSKL

>*Zm4CLL5*

MSIGAVVSPANPASTAEEYTHMVALSRPVVALAAPEVAAKLPRNLRCVVIGSNEYKSLSSAGGVAPSPPVAVKQSDTAAVLYSSGTTGRVKAAAVSHRNLIAVISAHRNNRVIAEKEAAEAGEEPLPPTVTLFPLPLFHVFGFMMLLRSVAMGETAVLMDRFDFIAALRAIERYRVTLLPAAPPVLVAMIKSEEARRCDLSSLLFIGIGGAPLGREVAERFAAIFPNIELIQGYGLTETSGSVASTVGPDECKAYGSVGKLASHLEAKIVDPSTGEVLGPGQRGELWVRGPVVMKGYVGDDEATAATVDSEGWLKTGDLCYFNEDGLLYIVDRLKELIKYKGYQVPPAELEHILNSHPDIMDAAVIPYPDEDAGQLPMAFIVRKPGSNLTEQQVMDFVAKQVAPYKKVRRVAFVSAIPKSPAGKILRRELVEQAVSMGTSKL

>*Zm4CLL4*

MAARPDARSGYCAATRTFRSKRADVPLPADADLDVVRFLASRRHAGVVALVDAATGHRVTFQELWRAVEGAATALAAPPLSLRKGQIALILSPNSVHFPVAALAAMSLGAVLTTANPLNTPAEIAKQVADARPVVAFTTRDLLPKLPRAGAGAGIRVVLLEPDRLPSDPSPVVATIGEISATPPDPTRRRDRVTQDDPATLLYSSGTTGPSKGVVATHRSLISMVQIIMSRFRLEASNRTETFLCTVPMFHVYGLVAFATGLLGCGATIVVLSKYELPEMLRAINEYGVTYLPLVPPILVAMLAYPNRLPLGGLRKVLSGGAPLSKELIEGFKEKYPQVEILQGYGLTESTAIGASTDSAEESSRYGTAGLLSPSTEAKIVDPETGEALPVNRTGELWIRGPYVMKGYFKNAEATQSTLTPDGWLKTGDLCYIDEDGYLFVVDRLKELIKYKGYQVPPAELEALLLTHPEIEDVTVIPFPDREVGQFPMAYVVRKKGSNLSEREVMEFVAKQVAPYKKVRKVAFVAEIPKNASGKILRKDLIKLATSKSKL

>*Zm4CLL7*

MVGTVSLAAGYGSDGVYRSPRLVAAIASDPGLSVTDLVLRRAAACPSAPALVDAATGRALTFGALRSAVLVAAAALSSRAGVRRGDAVLLLAPNCVLYPVCFLAVTALGAVATTANPLYTPREIAKQAADARARLVVTVSDLLPKISGLRLPIVLLDGDGAPVPSGCPSSNVTLYSDLVAGVQEAEYRHTPIGQGDTAALFYSSGTTGESKGVVLTHRNFIAAATMVTSDQDELGEGHNVFLCFLPMFHIFGMSVITLGQLQRGNAVVVMARFDVDAVLAAIERHRVTYLFCAPPAMIALAKHSRGGRYDLSSLRCIGSGAAPLGKDVMVAMADRFPGVLLLQGYGMTETCGIISLEYVQKGCARQFGSTGALVTGVEAKIVNAKTMKHLPPSQLGEICVRGPNIMEGYFNNVQATESTIKNGWLHTGDLGYFDERGQLHVVDRLKELIKYKGFQIAPAELEGLLLSHPEILDAAVIPYPDAEAGEVPIAYVVLSPKSSLAEADVQKFIEKQVKVGQKRQA

>*St4CL2*

MPMDIETKQSGDLIFRSKLPDIYIPKHLPLHSYCFENLSEFNSRPCLIDGANDRIYTYAEVELTSRKVAVGLNKLGIQQKDTIMILLPNCPEFVFAFIGASYLGAISTMANPLFTPAEVVKQAKASSAKIVITQACFAGKVKDYAIENDLKVICVDSAPEGCVHFSELIQSDEHEIPDVKIQPDDVVALPYSSGTTGLPKGVMLTHKGLVTSVAQQVDGENANLYMHSDDVLMCVLPLFHIYSLNSVLLCALRVGAAILIMQKFDIAQFLELIPKHKVTIGPFVPPIVLAIAKSPLVHNYDLSSVRTVMSGAAPLGKELEDAVRAKFPNAKLGQGYGMTEAGPVLAMCLAFAKEPFDIKSGACGTVVRNAEMKIVDPDTGCSLPRNQPGEICIRGDQIMKGYLNDPEATARTIEKEGWLHTGDIGFIDDDDELFIVDRLKELIKYKGFQVAPAELEALLINHPDISDAAVVPMIDEQAGEVPVAFVVRSNGSTITEDEVKDFISKQVIFYKRIKRVFFVETVPKSPSGKILRKDLRARLAAGISN

>*St4CL1*

MPMDTETKQSGDLIFRSKLPDIYIPKHLPLHSYCFENLSEFNSRPCLIDGANDRIYTYAEVELTSRKVAVGLNKLGIQQKDTIMILLPNCPEFVFAFIGASYLGAISTMANPLFTPAEVVKQAKASSAKIVITQACFAGKVKDYAIENDLKVICVDSVPEGCVHFSELIQSDEHEIPDVKIQPDDVVALPYSSGTTGLPKGVMLTHKGLVTSVAQQVDGENANLYMHSDDVLMCVLPLFHIYSLNSVLLCALRVGAAILIMQKFDIAQFLELIPKHKVTIGPFVPPIVLAIAKSPLVDNYDLSSVRTVMSGAAPLGKELEDAVRAKFPNAKLGQGYGMTEAGPVLAMCLAFAKEPFDIKSGACGTVVRNAEMKIVDPDTGCSLPRNQPGEICIRGDQIMKGYLNDPEATARTIEKEGWLHTGDIGFIDDDDELFIVDRLKELIKYKGFQVAPAELEALLINHPDISDAAVVPMIDEQAGEVPVAFVVRSNGSTITEDEVKDFISKQVIFYKRIKRVFFVETVPKSPSGKILRKDLRARLAAGISN

>*Md4CL2*

MISIASNSVETQKPADTSTNLMPPLINSTSQQNLTQLQPPACTNNIIDSTTATATINHVFRSKLPDIPIPNHLPLHTYCFQNLPEFSDRPCLIVGSTGKSYSFSETHLISQKTGAGLSNLGIQKGDVIMILLQNCAEFVFAFMGASLIGAVTTTANPFYTSAEIFKQVKAANAKLIITQSQYVDKLREHPSSADGADQNNYPKLGEDFKVVTIDDPPENCLHFSVLSEANENELPDVVIDAEDPVALPFSSGTTGLPKGVILTHKSLVTSVAQQVDGENPNLYLKEDDVVLCVLPLFHIFSLNSVLLCSLRAGAGVLLMHKFEIGTLLELIQRYRVSVAAVVPPLVIALAKNPMVAEFDLSSIRVVLSGAAPLGKELEEALKSRVPQALLGQGYGMTEAGPVLSMCMAFAKEPMPSKSGSCGTVVRNAELRVLDPETGLSLGYNQPGEICIRGSQFLPGYLNDAAATATTIDTEGWLHTGDVGYVDDDDEVFIVDRVKELIKFKGFQVPPAELESLLISHPSIADAAVVPQKDDAAGEVPVAFVVRSDGLELTEEAVKEFIAKQVVFYKRLHKVHFVHAIPKSASGKILRKDLRAKLATATPPLP

>*Md4CL2L*

MTIASSSVETQKPADIPTNLMPSEINSTSQQNLTQLQPAACTNNIIDSTTATSTATATTNHVFRSKLPDIPIPNHLPLHTYCFQNLPEFSDRPCLIVGSTGKSYSFSETHLIAQKTGAGLSNLGIQKGEVIMILLQNCAEFVFAFMGASMIGAVTTTANPFYTAAEVFKQVKAANAKLIITQSQYVNKLREHPSSADGTDQNNFPKLGEDFKIVTIDNPPENCLHFSVLSEANEKELPDVVIDAEDPVALPFSSGTTGLPKGVILTHKSLVTSVAQQVDGENPNLYLKEDDVVLCVLPLFHIFSLNSVLLCSLRAGAGVLLMHKFEIGTLLELIQRYRVSVAAVVPPLVIALAKNPMVAEFDLSSIRVVLSGAAPLGKELEEALKSRVPEAVLGQGYGMTEAGPVLSMCMAFAKEPMPTKSGSCGTVVRNAELKVLDLETGLSLGYNQSGEICIRGSQIMKGYLNDVAATATTVDTEGWLHTGDVGYVDDDNEIFIVDRAKELIKFKGFQVPPAELESLLISHPSIADAAVVPQKDDAAGEVPVAFVVRSNGLELTEEAVKEFIAKQVVFYKRLHKVHFVHAIPKSPSGKILRKDLRAKLATATPSALAN

>*Md4CLL9*

MAEESAANLRRWIDPKSGYCPRTKTFHSLRPPVPLPPLSQPLSLTQFTLSLLRSSMAKPTTSALIDAASDRHVSYQTFLSQIHSLTLSLQSLALFKGHVAFILSPPSLHVPVLYFSLLAFGVVVCPANPIGSESEIAHQVRLTRPAIAFATSATANKLPKGDDKIRTVILDSPEFLSMLDRDGSSVTRYESSVEVNQTDPAAILFSSGTTGRVKGVMLTHRNFIALLAGLHTLRREPDPTLLDGQPVSLFTLPLFHVFGFFMVVRAVALGETLVLMEKFQFEAMLRAVERHKVWYMPVSPPLIVALSKSELAQKYDLSSLRLLGCGGAPLGKEAADRFGERFPNVEVVQGYGLTETGGGATRMIDPEECARHASVGRLAENMEAKIVDPETGEALPPGKRGELWLRGPTIMKGYVRDDKATTETLDEDGWLKTGDLCYFDDEGFLYIVDRLKELIKYKAYQVPPAELEHILQSHPDVEDAAVIPYPDEDAGQIPMAYVVRKPGSNITEALVMDFVAKQVAPYKKIRRVAFINSIPKSPAGKILRRELVTHALSTGSSKL

>*Md4CLL5*

MANTPTVDARSGFCSSNSVFYSKRRSIPLPPNDSLDITTFISSQAHRGNVAFIDGSTGQQLTYVQLWDAVRSVASSLSDMGIRKGHVVLLLSPNSIFFPVVCLAVMSLGAIITTTNPLNTTREIAKQIADSKPVLAFTTRQLIPKLAGPIPLPIVLINDEIDAPIDNKSIVNTLGKMMAIKKHDGLKSREAIIQDDTATLLYSSGTTGASKGVVSSHRNLIAMVQTVIGRFYSDDNHHQTFLCTVPMFHIYGLVAFATGLLASGSTIVVLSKFEMHDMLLSIQRNRVSYLPLVPPILVALINAADQVKAKYDLSSLRWVLSGGAPLSKEVIEGFLEKYPMVSILQGYGLTESTGVGASTDSLEESRKYGTAGLLSASMEAMIVDPDSGHPLATNKTGELWLKGPTIMKGYFSNAEATASTLDSKGWLRTGDLCYIDEDGFIFVVDRLKELIKYKGYQVPPAELEALLLTHPEIADAAVIPFPDEKVGQYPMAYVVRNAGSSLTESGVMEFVGKQVAPYKRIRRVAFIDSVPKNPSGKILRKDLIQLAASSSSSSKL

>*Md4CLL6*

MAMTTLTKKFNNHISQKQSTQDGTPDHKYPWWFSPETGIYCSKHPEITLPTNHFLDVVTFIFSHQHTGVSPALIDSSSGSSLSYSQLYSFVKSFASGLRQMGISQGDVVLFLLPNSIYYPIVFLGVLYAGAIVTTMNPLSSVEEIKKQVGDSKACHAFTGLEYVGKLQALGVPAVLVPENAVLDSKNEAFSVFYKLVNGNVGLAPRPVIRQQDTAAIMYSSGTTGASKGVVITHRNFIAAVELFVRFEASQYEGSSLSDVYLDVLPLFHIYGLLLFVMGLFSLGSRIVVMKKFDVSEVVKAIEKYKVTHFPVVPPILTALTAKAKDGLAKQSLKSLKQVSSGAAPLSAKSIEDFVRTLPHVDLIQGYGMTETTAVGTRGFNTESLKKPSSIGLLAPNMQAKVVDWKTGSPLPPTSSGELWLRGPGIMNGYLNNADATMSTIDEEGWLHTGDIVYFDEDGYLYLQDRLKDAIKYKGFQIAPADLEGVLINHPEILDVAVAGVVDEESGEIPVAFVVRKHGSRILEDNVMDFVAAQVAPYKKVRKVVFTDSIPKSAAGKILRRELRKVLTSRL

>*Md4CLL7*

MEKSGFGRDGIFRSLRPPLVLPRDPNLSMVSFLFRNSSYSHKPALIDGESSETLSFSQFKSKVIQVSHGLINLGIKKNDVVLIFAPNSIQFPICLFGIIASGAIATTSNPLYTVSELSKQVRDSNPKLVITVPELWDKVKGFNLPTVFLGSTGFSGESHVGSASKIRTFHDLIESAGSVSDFPSVNIKQTDTAVLLYSSGTTGVSKGVVLTHRNFIASSLMVTMDQELSGEMHYVFLCVLPMFHVFGLAIITYGQLQKGNAVVSMGRFDLEKVLMAVEKYRVTHLWIVPPIVLALAKNSVVKKFNLSSLKHIGSGAAPLGKELMEECAKIIPQGVVAQGYGMTETCGIVSVENTLAGSRHTGSTGMLVSGVECQIVSVDTLKPQPPKQLGEIWVRGPNMMTGYFNNPQATNETIDKNGWVHTGDLGYIDEDGQLYVVDRIKELIKYKGFQVAPAELEGLLVSHPEILDAVVIPFPDAEAGEVPAAYCVRTPNSSLTEEDVKSFIASQVASFKRLRRVSFINSVPKSASGKILRRELIEKVRSKI

>*Md4CLL1*

MVNLIPSLVSTSLWLPLAPFHIFIMGTFVQNSAQEDEHIFGSRSPSVRVPDNITLPEFVLQNAELYAGNVAFVEAVTGKKITYGEVVRDTRRFAKALRSLGLRKGQVVVVVLPNVAEYAIVALGIMAAGGVFSGANPSGHASEIKKQVEVADAKIIVTNGATYEKVKSFGVPVILLGEEVIEGAMNWEHLLEAADRAGNRINEEELKQTDLCALPFSSGTTGLSKGVMLTHRNLVANLSSTLFGVTPEMIGKVTVLGLIPFFHIYGITGICCATLRNKGKVVVMGRYELRTFLNALISHEVTYAPIVPPIILNLVKNPIVDEFDLSKLKLQAIMTAAAPLAPEVLNAFENKFPGVQVQEAYGLTEHSCITLTHAGPGLVAKKNSVGFILPNLLVKFIDPDTGRSLPRNTPGELCVLSQCVMQGYFNNEEETARTIDKDGWLHTGDIGCIDDDDNVFIVDRIKELIKYKGFQVAPAELEAILLSHPSVEDVAVVPLPDEEAGEIPAASVVIAAGTKESEEEIINYVASNVAQYKKVRVVHFVDTIPKSPSGKIMRRLIKESMMEKIKASKLHCLN
