# Supplementary material for: Integration of metabolomics and transcriptomics reveals the regulation mechanism of the phenylpropanoid biosynthesis pathway in insect resistance traits in Solanum habrochaites
Source: Hortic Res. 2024 Jan 9;11(2):uhad277. doi: 10.1093/hr/uhad277 (PMC10857935; doi:10.1093/hr/uhad277)
Supplement: Web_Material_uhad277 [file web_material_uhad277.zip › Table S1 Primers for qRT-PCR analysis.doc]

Table S1 Primers for qRT-PCR analysis

| Gene name | Primer sequences (5'→3') |
| --- | --- |
| *PAL* primer | F: AAGGCCTTACATGGTGGCAA  R:TCCTACTGTCAGATTAGATGGCA |
| *4CL* primer | F: GTGGACTCAGCTTCTGGCAT  R: ACCAAGAGCCAAAACACCCA |
| *HCT* primer | F: GTGCCGTTTTATCCGATGGC  R: GTCGGACTCAGCTTCCACAA |
| *CAD* primer | F: TGGTTGGGCAGCTCATGATT  R: CCAGGCACAACAGGGTACTT |
| *UGT72E* primer | F: TGTGGGCCAATCAAGTCGAA  R: GGAACCCCATTCGTAAGGCT |
| *CHS* primer | F: TGTGTGCATACATGGCACCT  R: TTGGGCTGCCTCTTTTCCAA |
| *CHI* primer | F: GTTTCCCCTGCAGCAAAGTG  R: GGCGTTTCAGCACCAGAAAG |
| *F3H* primer | F: ACAACAAGCTATGCGCGTAAT  R: CCTCCGTAGGAAATGGCGTT |
| *MYB78* primer | F: TGGCGAACTCGGGTACAAAA  R: CGGCGGCTTGTATTCTTTCG |
| *AC actin* primer | F: AAGCATGAAGATACAGGGAGTGTG  R: ACATGTTGGAGAAGGCTCTTATTTAAA |
| *SH actin* primer | F: CGTGGTTATGTTGCCTCAAA  R: ACAGCAATGTGGGAAGTGTG |
